# A randomized, placebo-controlled, double blind Phase 1 trial of the Aβ vaccine, AV-1959R, in non-cognitively impaired participants: prelude for secondary preventive therapy in preclinical AD

**DOI:** 10.1186/s13024-026-00980-y

**Published:** 2026-07-22

**Authors:** Armine Hovakimyan, Hovhannes Madoyan, Zahra Najarzadeh, Garri Chilingaryan, Irina Petrushina, Grigor Mamikonyan, Ekaterina Lebedeva, Aleksandr Zaitsev, Kimiya Mansour, Thomas P. O’Malley, Shane Gonen, Roman Kniazev, Nikolai Petrovsky, Alexander Grinberg, Robert C. Alexander, Lon S. Schneider, Ravshan Ataullakhanov, Charles Glabe, Anahit Ghochikyan, Michael G. Agadjanyan

**Affiliations:** 1 Department of Molecular Immunology, Institute for Molecular Medicine, Huntington Beach, CA 92647 USA; 2 Nuravax Inc., Irvine, CA 92617 USA; 3 The Department of Molecular Biology and Biochemistry, University of California, Irvine, Irvine, CA 92697 USA; 4 Institute for Memory Impairments and Neurological Disorders, University of California, Irvine, Irvine, CA 92697 USA; 5 BostonGene, Corp., 95 Sawyer Road, Waltham, MA 02453 USA; 6 Department of Neurobiology & Behavior, University of California, Irvine, Irvine, CA 92697 USA; 7 Vaxine Pty Ltd, Flinders Medical Center, Bedford Park, Adelaide, 5042 Australia; 8 Arvax, Inc., Adelaide, Australia; 9 Banner Alzheimer’s Institute, Phoenix, AZ 85006-2727 USA; 10 Keck School of Medicine, University of Southern California, Los Angeles, CA 900330 USA

**Keywords:** Alzheimer’s disease, Prevention, Treatment, Phase 1, Clinical trial, β-amyloid, Vaccine, MultiTEP platform, Antibody titer

## Abstract

**Background:**

Active immunotherapy targeting amyloid-β (Aβ) represents a promising strategy for preventing Alzheimer’s disease (AD) in cognitively normal individuals at risk, referred to as the preclinical AD population. However, all Aβ vaccines tested so far in people with disease have faced significant challenges, primarily due to inadequate immunogenicity and insufficient antibody titers required to clear, reduce, or slow amyloid pathology. AV-1959R is a new generation MultiTEP-based Aβ vaccine specifically designed to overcome self-tolerance, provide broad T helper cell support, avoid the activation of Aβ-specific T cells, and elicit high levels of antibodies that selectively target pathological Aβ species in 100% of vaccinated individuals.

**Methods:**

We conducted a randomized, double-blind, placebo-controlled Phase 1 trial in 16 healthy adults aged 40–60 years. Participants received three intramuscular immunizations of AV-1959R (100–300 µg) or placebo formulated with Advax-CpG55.2 adjuvant (Day 1, Week 4, Week 14). Primary endpoints were safety and tolerability; secondary endpoints included humoral immunogenicity, antibody specificity, and isotype profiling. Antibody responses were assessed using validated ELISA and conformational Aβ microarray assays, and antibody kinetics were modeled to estimate durability.

**Results:**

AV-1959R was safe and well-tolerated, with no serious adverse events or ARIA observed. Adverse events were predominantly mild, transient injection-site reactions typical of adjuvanted vaccines. After receiving only two immunizations (priming), all vaccinated participants (12/12) developed antibody titers exceeding those reported for other Aβ vaccines in clinical trials. Induced antibodies selectively targeted pathological Aβ_42_ protofibrils and fibrils, with weaker binding to oligomers and minimal reactivity to monomers. Modeling based on priming and a single booster immunization predicts that antibody titers will remain 5 times above the baseline for approximately 1 year after priming, supporting an annual booster strategy. Vaccine-induced antibodies were of the IgG isotype and bound to amyloid plaques in human AD brain tissue.

**Conclusions:**

AV-1959R combines a favorable safety profile with potent, selective, and durable anti-Aβ immunity. Its 100% responder rate, induction of high antibody titers, selective targeting of pathogenic Aβ species, and dosing regimen distinguish AV-1959R from all previously discontinued Aβ vaccines and several AD vaccines tested in Phase 1/2 trial with Alzheimer’s and/or Down Syndrome individuals. These features classified AV-1959R as the top-tier, scalable immunogenic Aβ vaccine that can be safely used in the preclinical AD population with the evidence of amyloid pathology, with or without tau pathology, aiming to clear, reduce, prevent, or at least slow Aβ accumulation and the onset of Alzheimer’s. The protocol for a planned Phase 2 preventive trial of AV-1959R under IND 30925-0004, has received IRB approval. Once initiated, the study will evaluate the vaccine’s biological efficacy using established AD brain and blood biomarkers.

**Trial registration:**

Australian New Zealand Clinical Trials Registry (ANZCTR) ACTRN12624000737538; ClinicalTrials.gov NCT06831812.

**Supplementary Information:**

The online version contains supplementary material available at https://doi.org/10.1186/s13024-026-00980-y.

## Background

Alzheimer’s disease (AD) is a complex, multifactorial disorder characterized by the accumulation of amyloid-β (Aβ) oligomers, fibrils, plaques, intraneuronal tau aggregates and tangles, inflammation, and neurodegeneration. Genetic evidence from autosomal-dominant AD (APP and PSEN1/2 mutations) and the high prevalence of Alzheimer’s neuropathology in Down syndrome (trisomy 21 with APP gene triplication) strongly supports the amyloid cascade hypothesis [1–3]. Toxic Aβ oligomers are thought to initiate downstream pathological processes, including tau pathology, oxidative stress, neuroinflammation, and synaptic and neuronal loss [4–6]. Even subthreshold Aβ accumulation, detected before brain-Aβ positivity, correlates with subtle memory impairment and predicts cognitive decline [7]. Longitudinal studies demonstrate that although Aβ pathology emerges more than a decade before tau pathology, it is the interaction between Aβ and phosphorylated tau (p-tau) that drives the progression from mild cognitive impairment (MCI) to dementia [4, 8–10]. Accumulating genetic, biomarker, and therapeutic evidence strongly supports targeting pathological Aβ to halt/modify disease progression [11]. This paradigm has been supported by FDA and EMA marketing approval of the anti-Aβ monoclonal antibodies (mAbs) lecanemab (Leqembi^®^) and donanemab (Kisunla™), which significantly reduce brain amyloid plaques and modestly attenuate clinical decline in individuals with early symptomatic AD [12–15]. However, the use of amyloid-targeting mAbs for preventing cognitive impairment in individuals at risk for AD is impractical. Monoclonal antibody treatment would have to be administered intravenously or subcutaneously at 2- to 4-week intervals for several years during the preclinical stage [16, 17]. Moreover, the current mAbs and those in phase 2 and 3 clinical development are associated with high rates of amyloid-related imaging abnormalities (ARIA), i.e., vasogenic edema (ARIA-E), microhemorrhages (ARIA-H), and superficial siderosis, particularly in individuals who carry the APOE ɛ4 allele (coding for apolipoprotein E4) and individuals with cerebral amyloid angiopathy [15, 16, 18, 19]. An immunogenic Aβ-vaccine would serve as a more efficient and effective prophylactic modality for preventing cognitive impairment due to Alzheimer’s pathology. To be effective, it should elicit high-titer antibodies against Aβ and halt or attenuate the buildup of amyloid plaques, an upstream process that contributes to subsequent tau pathology, thereby delaying or preventing the onset of clinical disease.

Here we report the first-in-human evaluation of AV-1959R, a MultiTEP-based Aβ vaccine [20, 21] formulated with Advax-CpG55.2 adjuvant [22, 23]. The AV-1959R is engineered to overcome self-tolerance without activating autoreactive T cells, activate pre-existing memory T helper cells, and provide broad MHC class II coverage. It has demonstrated robust safety, efficacy, and immunogenicity in preclinical IND-enabling studies [21, 24–29]. The Advax-CpG55.2 adjuvant, which combines delta inulin with the TLR9 agonist CpG55.2, has been used to enhance the vaccine-induced anti-Aβ antibody response [20]. The primary objective of this randomized, placebo-controlled, double-blind Phase 1 study was to assess the safety and tolerability of AV-1959R, and the secondary objective was to evaluate and characterize humoral immune responses, including anti-Aβ antibody levels, specificities, and their isotypes/subtypes, in individual participants’ sera.

## Methods

### Ethics

This study was conducted at CMAX Clinical Research, Adelaide, South Australia, in accordance with the study protocol, the ethical principles derived from international guidelines, including the Declaration of Helsinki, the International Ethical Guidelines of the Council for International Organizations of Medical Sciences (CIOMS), applicable ICH GCP Guidelines, and other applicable laws and regulations. The protocol was approved by the Bellberry Human Research Ethics Committee (Eastwood, South Australia).

### Trial registration

This randomized, placebo-controlled, double-blind Phase 1 study was prospectively registered with the Australian New Zealand Clinical Trials Registry (ANZCTR; ACTRN12624000737538) prior to enrollment of the first participant. The study was also registered on ClinicalTrials.gov (NCT06831812).

### Study design

This was a single-center, phase 1, randomized, double-blind, placebo-controlled, multiple-ascending-dose trial conducted in healthy male and female participants to evaluate the safety, tolerability, and immunogenicity of the anti-Aβ vaccine AV-1959R adjuvanted with Advax-CpG55.2 (ACTRN12624000737538 and NCT06831812).

Participants were enrolled into one of two sequential dose cohorts and received three immunizations of either 100–300 µg of the AV-1959R vaccine or placebo, each formulated with the Advax-CpG55.2 adjuvant. The study duration was 26 weeks, including a 4-week screening period, a 14-week treatment period, and an 8-week follow-up period. Written informed consent was obtained from all participants prior to enrollment.

### Participants

Sixteen healthy male and female participants of non-childbearing potential, either medically sterile or using adequate contraception, aged 40 to 60 years, were enrolled in this trial. Other inclusion criteria were: body mass index (BMI) between 18.0 and 32.0 kg/m² at the screening visit; absence of clinically significant medical history; no abnormalities on physical and neurological examinations, 12-lead ECG, MRI, blood or urine tests; and no use of medications that could interfere with the IP or study outcomes.

### Eligibility criteria

#### Inclusion

Eligible participants are healthy adults aged 40–60 years with a BMI of 18–32 kg/m², without clinically apparent cognitive impairment, and with no clinically significant findings on medical history, physical exam, laboratory tests, ECG, or MRI at Screening/Baseline. Although formal cognitive testing or screening was not performed because it was not part of the study protocol, participants were assessed for their ability to provide informed consent, and no cognitive impairments were identified during the Principal Investigator’s clinical assessment and overall screening evaluation.

Participants must provide written informed consent and comply with study requirements.

Women must be of non-childbearing potential, defined by postmenopausal status or documented surgical sterilization. Men must be surgically sterile or agree to appropriate contraceptive measures for the required duration.

#### Exclusion

Key exclusions include any clinically significant medical condition or MRI abnormalities (including lacunar or territorial infarcts, microbleeds, leptomeningeal hemosiderosis, ARIA-E, or other major structural findings). Individuals with contraindications to MRI, serious illness or hospitalization within 4 weeks, significant cardiovascular, respiratory, gastrointestinal, endocrine, hematologic, immunologic, or other systemic disorders are excluded. Additional exclusions include recent major surgery, hypersensitivity reactions, substance or alcohol abuse, and clinically significant laboratory abnormalities (including abnormal blood counts, elevated liver enzymes, impaired kidney function, or positive HIV, HBV, or HCV tests).

Participants with abnormal vital signs (marked hypertension or hypotension, bradycardia or tachycardia), prolonged QTcF, or any condition compromising safety or study compliance are excluded. Other exclusion criteria include recent participation in another investigational study, prior tau or amyloid-beta immunotherapy, use of immunomodulatory agents or chronic anticoagulant/antiplatelet therapy, administration of blood products, severe prior vaccine reactions, pregnancy/childbearing potential in females, skin conditions or tattoos interfering with injection-site evaluation, and recent significant blood loss or donation. APOE genotyping was not performed in this Phase 1 study and was therefore not used as an inclusion or exclusion criterion.

### Randomization

Eligible participants were randomly assigned in a 3:1 ratio to receive injections of either AV-1959R or placebo, both formulated with adjuvant, according to a randomization sequence generated using the Statistical Analysis System (SAS) software version 9.4. Dosing within each cohort was staggered. Two participants (one receiving AV-1959R and one receiving a placebo) received the first two immunizations (Immunizations 1 and 2) before the remaining participants of the cohort. After a two-week safety review confirmed acceptable tolerability, the remaining cohort participants (five receiving AV-1959R and one receiving placebo) proceeded with their scheduled immunizations.

### Blinding

The study participants, investigators, sponsor, and all staff members involved in the conduct of the study were blinded to the treatment assignment. The randomization schedule was generated by an unblinded statistician and stored in a secure, locked area accessible only to the assigned unblinded pharmacist or designee. To preserve the study blind, the vaccine and placebo were identical in appearance.

Each dose of adjuvanted AV-1959R or placebo was prepared and dispensed by an unblinded pharmacist in accordance with the randomization schedule and then transferred to the trial clinical site on the day of immunization. Dosing was performed by the trained clinical site personnel. All participants received the assigned intervention as planned; no deviations in dosing or administration were reported.

### Procedures

All participants were screened within 4 weeks before administration of the study drug. Eligible participants were admitted to the study site the day before dosing. Immunizations were given on Days 1, 28±2, and 98±5, with follow-up visits 2 weeks after each dose (Days 14±2, 42±3, and 112±5) and during the follow-up period (Days 140±5 and 154±7). Dose escalation for the second cohort was based on the Safety Review Committee (SRC) review of safety data through 14 days after the first immunization of all eight participants in the first cohort.

AV-1959R is a recombinant protein composed of three copies of the N-terminal region of human Aβ and an immunogenic vaccine platform composed of 12 foreign promiscuous T helper (Th) cell epitopes [one synthetic epitope and 11 epitopes from several microorganisms (tetanus toxin; hepatitis B and influenza viruses)] collectively designated as the MultiTEP vaccine platform [20, 21].

To enhance the immunogenicity of AV-1959R, the Advax-CpG55.2 (Vaxine Pty. Ltd) adjuvant was used in this trial.

AV-1959R was supplied by the sponsor in sterile vials containing 0.84 mL at 1.2 mg/mL, with a total content of 1.01 mg. Adjuvant Advax-CpG55.2 was supplied in two separate vials containing 0.5 mL at 50 mg/mL, total content 25 mg, and 0.5 mL at 2 mg/ml, total content 1 mg, respectively. The final investigational product was prepared in the dedicated pharmacy by sequentially mixing Advax, CpG55.2, and normal saline with AV-1959R and contains 15 mg Advax/0.15 mg/CpG55.2 and 100 µg or 300 µg AV-1959R. The placebo consisted of Advax, CpG55.2, and normal saline only. All injections were administered within 4 h of preparation.

Blood samples for anti-Aβ antibody titers were collected at baseline and on Days 42, 98, 112, 140, and 154.

### Safety assessments

Clinical laboratory tests and vital signs were reviewed for potential adverse findings. All events were coded using MedDRA version 28.0 and graded for severity using the Common Terminology Criteria for Adverse Events (CTCAE v5.0). Investigators and a medical monitor assessed the causality of each event as related, possibly related, or unrelated to study treatment. Adverse event data were collected from the ICF signature through Day 154 (end of the follow-up) of the study.

If a predefined safety criterion was met, the study would be paused for review by the SRC. All safety data were summarized descriptively.

### Detection of Aβ-specific antibodies

The titers of vaccine-induced antibodies were determined using a validated ELISA performed under fully blinded conditions with respect to sample identity. Lyophilized Aβ_42_ peptide (Shanghai RoyoBiotech CO., LTD) was dissolved in Coating buffer B (50 mM carbonate buffer, pH 9.4), yielding a mixture of prefibrillar and fibrillar species (Fig. 2A), and was immobilized on microtiter plates (Immulon 2 HB plates, Thermo Fisher Scientific) at a final concentration of 10 µg/mL. Plates were blocked with Assay Buffer (1X, Antibody Pair Buffer Kit, Invitrogen by Thermo Fisher Scientific). Serial dilutions of serum samples (1:100 to 1:204,800) were added to the plates, and bound antibodies were detected using Peroxidase AffiniPure™ F(ab’)₂ Fragment Goat Anti-Human IgG, Fcγ fragment-specific secondary antibody (Jackson ImmunoResearch), diluted 1:5,000 in Assay Buffer (1X). After washing, bound secondary antibodies were visualized using the Stabilized Chromogen (TMB) provided in the same kit, and the reaction was stopped with the supplied stop solution. Optical density (OD) was measured at 450 nm using a FilterMax F5 plate reader. Antibody titers were defined as the reciprocal of the highest serum dilution with an OD₄₅₀ ≥ cutoff (C), corresponding to the lower limit of quantification (LLOQ = 0.274), calculated as 3.3 × the lower limit of detection (LLOD).

To ensure assay accuracy and consistency, a reference standard curve was generated using serial dilutions of the monoclonal anti-Aβ antibody aducanumab, which recognizes the N-terminus of Aβ, starting at 50 ng/mL. The humanized anti-HER2 antibody trastuzumab served as a negative control. Positive and negative control sera were included on each plate to ensure assay consistency.

Assay recovery was assessed by spiking aducanumab into four independent human serum samples, resulting in an average recovery of 109%, confirming assay accuracy and precision in a serum matrix.

### Calculation of antibody titers

Exact antibody titers were determined from ELISA data by interpolation between the two consecutive serum dilutions flanking the cutoff OD value, set at 3.3 × the lower limit of detection (LLOD). The LLOD was calculated as the mean OD of blank wells plus three times the standard deviation (SD) of the blank, based on data from ≥ 10 independent assay runs.

The titer (T) was calculated using the linear interpolation formula:

$$\:T={D}_{1}+\frac{C-{R}_{1}}{{R}_{2}-{R}_{1}}\times\:\left({D}_{2}-{D}_{1}\right)$$

where

- D₁ = dilution corresponding to the last positive OD (e.g., 400),
- D₂ = dilution corresponding to the first negative OD (e.g., 800),
- C = cutoff (OD = 0.274),
- R₁ = OD for D₁ (e.g., 0.300),
- R₂ = OD for D₂ (e.g., 0.200).

The resulting titer represents the reciprocal dilution at which the OD₄₅₀ equals the cutoff. This interpolation method provides greater precision than the endpoint dilution approach and ensures assay comparability across runs and plates.

The isotypes (total IgG, IgM, IgA, and IgE) of anti-Aβ antibodies were evaluated in pooled serum samples collected on Days 1, 42, and 112, each diluted 1:3,200 for every cohort. IgG subclasses were analyzed in individual serum samples collected on Days 1 and 42, diluted 1:200. The following horseradish peroxidase (HRP)-conjugated secondary antibodies were used to detect specific isotypes and IgG subclasses of serum antibodies: AffiniPure™ F(ab’)₂ Fragment Goat Anti-Human IgG, Fcγ fragment-specific antibody (Jackson ImmunoResearch), at dilution of 1:5,000; Mouse Anti-Human IgM (Heavy Chain) Antibody (Thermo Fisher Scientific) at 1:800; Goat Anti-Human IgA Antibody (Thermo Fisher Scientific) at 1:6,000; Mouse Anti-Human IgE Antibody (Thermo Fisher Scientific) at 1:750; Mouse Anti-Human IgG1 Fc Antibody (Thermo Fisher Scientific) at 1:1,250; Mouse Anti-Human IgG2 Antibody (Thermo Fisher Scientific) at 1:800; Mouse Anti-Human IgG3 (Hinge) Antibody (Thermo Fisher Scientific) at 1:800; and Mouse Anti-Human IgG4 Antibody (Thermo Fisher Scientific) at 1:38,000. The OD value was measured at 450 nm (FilterMax F5).

### Preparation of Aβ_42_ monomer, oligomer, protofibril, and fibril

#### Aβ_42_ monomer preparation

Aβ_42_ synthetic peptide (1 mg) was dissolved in 300 µL of hexafluoroisopropanol (HFIP) and sonicated for 1 cycle (30 s ON, 30 s OFF) using a Bioruptor bath sonicator (Diagenode). The HFIP was evaporated under a nitrogen gas stream, and this process was repeated three times. Residual HFIP was removed using a speed vacuum. The resulting monomer layer was dissolved in dimethyl sulfoxide (DMSO) to 1 mM. The monomer was then diluted in ice-cold 100 mM NaOH to the desired concentration and used immediately for spotting.

#### Aβ_42_ oligomer preparation

Aβ_42_ peptide (1 mg) was dissolved in 300 µL HFIP as described for monomer preparation. The monomer layer was dissolved in 45 µL of 10% (v/v) NH_4_OH and diluted with 885 µL of ice-cold PBS (pH 12). The solution was sonicated for 2 cycles and centrifuged at 14,000 × g for 10 min at 4 °C. The supernatant was ultracentrifuged at 70,000 rpm using a TLA110 rotor for 1 h at 4 °C. The resulting supernatant was transferred to a new tube, and 10 µL of 10 N NaOH was added per 100 µL of supernatant. The solution was then diluted to the desired concentration with 10% (v/v) NH_4_OH, sonicated on ice for 4 cycles, and kept on ice until use. This method produces “prefibrillar oligomers” that react specifically with the A11 antiserum and monoclonal antibodies derived from A11-producing rabbits. Prefibrillar oligomers are structurally related to membrane-forming pores and immunologically distinct from protofibrils and other oligomers structurally related to fibrils [30]. Oligomer formation was verified by atomic force microscopy (AFM).

#### Aβ_42_ protofibril preparation

Aβ_42_ peptide (0.3 mg) was dissolved in 51 µL of 50 mM NaOH and sonicated for 2 cycles using a Bioruptor bath sonicator. The solution was centrifuged at 14,000 × g for 10 min to remove undissolved peptide. The supernatant was diluted in 20 mM phosphate buffer (PB, pH 7.5) to a final concentration of 400 µM and incubated at room temperature under quiescent conditions for 24 h. Protofibril formation was confirmed by AFM.

#### Aβ_42_ fibril preparation

After HFIP treatment, as described for monomer preparation, the Aβ_42_ peptide film was dissolved in 50 mM NaOH to a final concentration of 1.3 mM, then diluted in 20 mM phosphate buffer (pH 7.4) to a final concentration of 100 µM. The solution was then incubated at 37 °C with shaking at 200 rpm for 7 days to allow fibril formation.

#### Aβ microarrays

Freshly prepared Aβ_42_ species were used to prepare microarrays. A total of 50 µL from each sample was dispensed into a 384-well plate (sciSOURCEPLATE 384 PS) and subsequently spotted onto nitrocellulose-coated glass AVID slides (Grace Bio-Labs, Inc., OR, USA) using a Scienion S3 sciFLEXARRAYER (Scienion AG, Berlin, Germany) operated in automated, piezo-driven, non-contact dispensing mode.

### Microarray incubation with individual and pooled sera

#### Individual serum incubation

Individual microarray pads were isolated using a 16-well multi-chamber tray placed on top of the slide and secured with metallic clamps. Each pad was incubated with 100 µL of Protein Array Blocking Buffer (PABB, GVS FAST™) for 30 min at room temperature, followed by aspiration. Individual sera were diluted 1:100 in PABB and added to each pad. Slides were incubated overnight at 4 °C with mild agitation. After incubation, the samples were aspirated, and nitrocellulose pads were washed three times with 100 µL of TBS-T (TBS + 0.05% Tween-20), each wash lasting 5 min. The secondary antibody (Goat anti-Human IgG-Alexa 647) was diluted 1:200 in PABB, and 100 µL was added to each pad. Slides were incubated at room temperature for 1 h with mild agitation. Pads were then washed three times with 100 µL of TBS-T, followed by centrifugation at 1,000 × g for 5 min to dry. Microarray pads were imaged and quantified using the Scionion sciREADER FL2 system. In parallel, microarrays were incubated with the positive control antibody 6E10, which binds to all Aβ conformers, to confirm that equal amounts of each conformer were spotted onto the nitrocellulose membrane (Fig. 3B).

### Atomic force microscopy

Samples were deposited onto freshly cleaved mica and allowed to adsorb for 10 min. The mica surfaces were then rinsed with deionized water, and the samples were air-dried overnight. Imaging was performed using TAP150Al-G soft tapping-mode AFM cantilevers (BudgetSensors) with a resonance frequency of 150 kHz and a force constant of 5 N/m. Images were acquired at a resolution of 1024 × 1024 pixels in tapping mode using an HR-AFM system (AFM Workshop, USA) and analyzed with Gwyddion 2.67.

### Negative-stain electron microscopy (EM)

Samples were prepared for negative stain utilizing a slightly modified version of a previously established protocol [31]. In brief: Gilder 200-mesh copper grids (Ted Pella) were carbon-coated using a Leica ACE200 sputter coater. Grids were negatively glow-discharged in a PELCO easiGlow for 45 s at 15 mA and 0.45 mBar. 3µL of 100µM sample was applied and allowed to adsorb for ~ 15 s, then blotted on a piece of filter paper. The grid was then washed twice with 50µL drops of Milli-Q water and stained with two 50µL drops of freshly prepared 0.75% Uranyl Formate. Excess stain was blotted with filter paper, and the grid was dried under vacuum. All grids were imaged using a JEOL-JEM 2100 F 200 kV transmission electron microscope equipped with a Gatan OneView 4k x 4k camera. Scale bars were added, and micrographs were contrast-enhanced using Fiji [32].

### Competition ELISA

Serial dilutions of Aβ_1-11_ (0.02 µM, 2.5 µM, 20 µM, 60 µM, 150 µM, 200 µM, and 300 µM) (Genscript Biotech) and Aβ_42_ (0.02 µM, 2.5 µM, 20 µM, 60 µM) peptides were incubated with pooled immune serum (Day 42) from both cohorts diluted 1:10,000 in assay buffer. Following preincubation with the competing peptides, serum binding to immobilized Aβ_42_ was assessed by standard ELISA as described above. The percentage of binding of sera preincubated with Aβ_42_ or Aβ_1-11_ peptides was calculated relative to sera without competing peptides, which was set as 100%. The half-maximal inhibitory concentration (IC50) was determined for each peptide.

### Aβ-specific antibody purification from pooled immune sera

Aβ-specific antibodies were affinity-purified from pooled Day 42 immune sera of vaccinated participants following the manufacturer’s instructions for the SulfoLink™ Immobilization Kit for Peptides, 2 mL (ThermoFisher Scientific, #44999). An Aβ_1-11_-C peptide (Genscript Biotech), the N-terminal Aβ_1–11_ sequence with an added C-terminal cysteine residue to enable immobilization on a SulfoLink column, was used at a concentration of 0.5 mg per column. The concentration of purified antibodies in the eluates was measured using the BCA method.

### Dot Blot

To evaluate the binding of Aβ-specific affinity-purified antibodies to Aβ_42_ protofibrils/fibrils and the pE_3_-Aβ₁₋₁₁ peptide (Genscript Biotech), 1 µL (1 µg) of each antigen was spotted onto nitrocellulose membranes (GE Healthcare, Piscataway, NJ). Membranes were blocked with 5% non-fat dry milk in TTBS and washed prior to incubation with vaccine-induced Aβ-specific affinity-purified antibodies. Commercial monoclonal antibodies Lecanemab and Donanemab were included as comparators, and Trastuzumab served as a negative control; all antibodies were used at equal concentrations. Following primary antibody incubation, membranes were probed with Peroxidase AffiniPure™ F(ab’)₂ Fragment Goat Anti-Human IgG (Fcγ-specific) secondary antibody (1:5000; Jackson ImmunoResearch, #109-036-098). Signal was developed using luminol reagent (Santa Cruz Biotechnology, #sc-2048) and detected by exposure to X-ray film (Kodak, X-Omat AR, Rochester, NY).

### Model for antibody kinetics

To describe the longitudinal kinetics of vaccine-induced antibody titers, we implemented a simplified bi-exponential decay model based on the framework reported in Andraud et al. [33]. This mathematical model is used to analyze how antibody levels change over time after vaccination, specifically to decompose the immune response into short-term and long-term components. This helps distinguish between the fast antibody surge in the first days/week(s) after vaccination, driven by short-lived plasma cells, and more stable, enduring antibody production driven by long-lived plasma cells.

Antibody titers $\:A\left(t\right)$ at time * t* were modeled as the sum of two exponential components:

$$\:A\left(t\right)=\:{B}_{1}\times\:{e}^{\left\{-{k}_{1}t\right\}}+\:{B}_{2}\times\:{e}^{\left\{-{k}_{2}t\right\}}$$

where $\:{B}_{1}$ and $\:{B}_{2}$ represent the contributions from short-lived and long-lived plasma cell compartments, respectively, while $\:{k}_{1}$ and $\:{k}_{2}$ are the corresponding decay rate constants.

Parameter estimation was performed using data from 12 donors, each with three timepoints (days 112, 140, 154, second peak) after boosting vaccination. Donor-specific parameters $\:{B}_{1}$ and $\:{B}_{2}$ were fitted individually, while the decay rate constants $\:{k}_{1}$ and $\:{k}_{2}$ were constrained to be identical across all donors, reflecting a shared kinetic profile of short- and long-lived plasma cell populations.

Model fitting was carried out via nonlinear least squares regression, minimizing the residual sum of squares between observed and predicted antibody titers. This approach allows the separation of inter-individual differences in the magnitude of antibody responses (captured by $\:{B}_{1}$ and $\:{B}_{2}$ from the common decay dynamics (captured by $\:{k}_{1}$ and $\:{k}_{2}$).

After estimating common values of $\:{k}_{1}$ and $\:{k}_{2}$, the model was subsequently applied to two timepoints (days 42, 98) for the first peak (priming). For these timepoints $\:{k}_{1}$ and $\:{k}_{2}$were fixed to the previously obtained values, and donor-specific coefficients $\:{B}_{1}$ and $\:{B}_{2}$ were obtained by solving a linear equations system. The estimated value of $\:{k}_{1}$ is 0.059, and for $\:{k}_{2}$ is 0.0062. Supplementary Table 3 contains the obtained parameters for short-term and long-term components ($\:{B}_{1}$ and $\:{B}_{2}$) for each donor. Supplementary Table 4 contains the estimated number of days to reach an antibody titer of 1:1000 after priming vaccination. To facilitate biological interpretation of the model-derived decay parameters, half-lives ($\:{t}_{1/2}$) for the short- and long-lived antibody components were calculated from the estimated rate constants $\:{k}_{1}$and $\:{k}_{2}$obtained in the bi-exponential model. Half-life was determined according to the exponential decay relationship.

$\:{t}_{1/2}=\frac{\mathrm{l}\mathrm{n}\left(2\right)}{k}$where $\:k\:$ represents the decay rate constant (per day) for the corresponding component. Using the fitted values $\:{k}_{1}$ = 0.059 day and $\:{k}_{2}$ =0.0062 day, the calculated half-lives were approximately 11.7 days and 111.8 days, respectively. These values represent the characteristic times required for the short- and long-lived plasma-cell-derived antibody fractions to decline by 50%.

### Detection of immune sera binding to Aβ plaques by IHC

*Mouse brain sections* were collected from 8-month-old bigenic T5X (Thy22 × 5xFAD) mouse model [21] of AD and their littermates. Pooled sera were collected from participants on day 42 of the trial, and the binding to Aβ plaques was assessed in mouse brain sections sliced after brain perfusion and fixation in 4% paraformaldehyde (PFA) for 24 h. Before immunostaining, tissues were stored in PBS containing 0.05% sodium azide.

Briefly, fixed half-brains were cryoprotected in 20% sucrose for at least 48 h before coronal sectioning (40 μm) on a freezing sliding microtome. Prior to IHC, free-floating sections were washed in Tris buffer, mounted onto SuperFrost™ Plus Slides (Epredia™), and air-dried overnight at room temperature. The following day, tissues were rehydrated in Tris buffer and subjected to epitope retrieval with 90% formic acid for 4 min at room temperature. Sections were then incubated overnight with pooled immune sera at a 1:500 dilution or with an irrelevant control antibody (trastuzumab, 2 µg/mL). The next day, washed slides were incubated for 1 h with a biotin-SP–affinity–purified donkey anti-human IgG Fcγ fragment-specific secondary antibody (Jackson ImmunoResearch) diluted 1:400 in Tris B buffer containing 4% normal donkey serum. Following the washing step and quenching of endogenous peroxidase activity, bound antibodies were visualized via Vectastain^®^ Elite^®^ ABC Kit (Vector Laboratories) according to the manufacturer’s instructions.

*Human brain tissues* were obtained from the Brain Bank and Tissue Repository, MIND ADRC, University of California, Irvine. Pooled sera from participants collected on day 42 of the trial were tested for IgG antibody binding to Aβ plaques in brain sections from an AD case (39 − 10). As a control, brain sections from a non-AD case (19 − 13) were used. Formalin-fixed hippocampal Sect.  (40 μm) were mounted on glass slides and air-dried. Sections were pretreated with 90% formic acid to enhance antigen retrieval, and endogenous peroxidase activity was quenched. Slides were incubated with pooled immune sera (diluted 1:50) or a negative-control irrelevant mAb (trastuzumab at 2 µg/mL) for 1 h. To visualize bound antibodies, the Human-on-Human (H.O.H) detection kit (Vector Laboratories, Burlingame, CA) was used according to the manufacturer’s instructions. After washing, the diluted HRP-conjugated secondary antibody was applied and incubated for 15 min. Following the second wash, the components of the peroxidase substrate solution were applied to the sections for 5 min to develop the desired stain.

### Analyses of whole-slide imaging

Whole-slide imaging was performed at the Alafi Neuroimaging Laboratory, Washington University School of Medicine (St. Louis, MO). Slides were digitized using a NanoZoomer 2.0 HT scanner (Hamamatsu, Bridgewater, NJ), and digital images were analyzed for immunoreactivity.

### Statistical analysis

The sample size of 16 subjects lacks a formal statistical basis and was chosen pragmatically, based on clinical considerations, to provide sufficient data to meet the safety objectives of this study. No software was used for sample size calculation. Trends and variability were assessed using descriptive statistics and individual-level data. Baseline, demographic, and safety variables were summarized descriptively. Anti-Aβ antibody titers were analyzed at each time point and summarized using descriptive statistics (i.e., n, mean, geometric mean, min, max, SD, CV) for each cohort, treatment group, and dose level.

#### Correlation analysis

Pairwise associations between extracted ELISA-derived features were assessed using linear regression. Goodness-of-fit was evaluated with the coefficient of determination (R²), while correlation strength and statistical significance were quantified using the Pearson correlation coefficient (r) and its corresponding p-value.

#### Software and statistical analysis

Data preprocessing, statistical analysis, and curve fitting were performed using pandas, numpy, and SciPy Python libraries. Visualization of results was performed using matplotlib and seaborn, including scatter plots, boxplots. Statistical differences between groups were assessed using the Mann–Whitney U test (two-sided), p-values were reported on the plots, and levels of significance were indicated with conventional markers (*p < 0.05, **p < 0.01, ***p < 0.001).

## Results

### Study participants

**Fig. 1:**
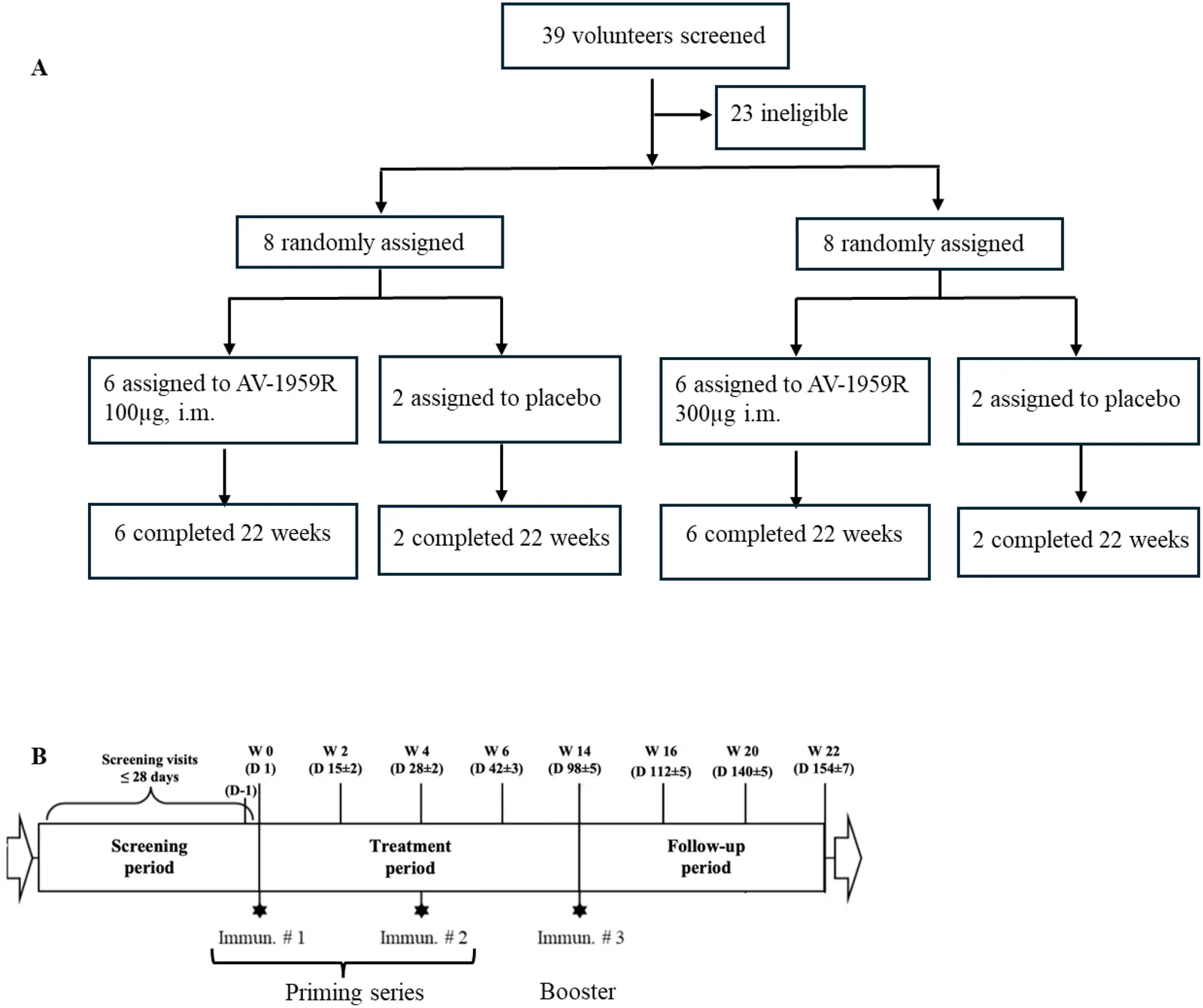
A Phase I, randomized, double-blind, placebo-controlled study to assess the safety, tolerability, and immunogenicity of AV-1959R in participants without clinically apparent cognitive impairment. (**A**) Trial profile. (**B**) Study design scheme

A total of 39 volunteers were screened, and 16 healthy male (*n* = 12) and female (*n* = 4) participants, aged 40–60 years, were enrolled between September 27, 2024, and February 4, 2025. Twenty-three participants were screen-failed due to not meeting inclusion/exclusion criteria (*n* = 16), withdrawal of consent (*n* = 3), being outside the screening window (*n* = 3) or completion of enrollment (*n* = 1) (Fig. 1A). Remaining 16 participants were randomized into one of two sequential cohorts in a 3:1 ratio to receive intramuscular injections of either AV-1959R (*n* = 12) or placebo (*n* = 4), both formulated with Advax-CpG55.2 adjuvant at baseline and week 4 (e.g., priming immunizations), followed by a booster injection at week 14, and were followed for safety up to week 22 (Fig. 1B).

Baseline characteristics were generally similar across the cohorts (Table 1), with mean age of 51.6 (range, 43–60) years and mean BMI of 27.3 (range, 22.1 to 31.3) kg/m^2^. 75% of participants were male and 25% were female; the racial composition was 100% white. The study achieved 100% completion, with all randomized participants included in the safety and immunogenicity analyses populations.

**Table 1:** Baseline characteristics of trial participants

Characteristic	All participants	Cohort 1–100 µg		Cohort 2–300 µg	
		AV-1959R	Placebo	AV-1959R	Placebo
N	16	6	2	6	2
**Age (years)**					
Mean (SD)	51.6 (4.77)	52.0 (5.02)	51.0 (2.83)	52.2 (4.71)	49.5 (9.19)
Min / Max	43/60	46/60	49/53	46/58	43/56
**Gender**,** n (%)**					
Male	12 (75.0)	5 (83.3)	1 (50.0)	5 (83.3)	1 (50.0)
Female	4 (25.0)	1 (16.7)	1 (50.0)	1 (16.7)	1 (50.0)
**Race**,** n (%)**					
White	16 (100.0)	6 (100.0)	2 (100.0)	6 (100.0)	2 (100.0)
**Height (cm)**					
Mean (SD)	176.94 (8.264)	177.4 (6.376)	169.35 (0.495)	179.5 (8.037)	175.5 (18.385)
Min / Max	162.5/194.2	169.8/186	169/169/7	171/194.2	162.5/188.5
**Weight (kg)**					
Mean (SD)	85.49 (9.22)	84.08 (8.231)	81.30 (2.121)	87.40 (11.643)	88.15 (13.081)
Min / Max	68.3/100.3	76.8/99.7	79.8/82.8	11.643	78.9/97.4
**BMI (kg/m** ^**2**^ **)**					
Mean (SD)	27.303 (2.2598)	26.703 (1.9008)	28.345 (0.5728)	27.108 (3.0658)	28.645 (1.7466)
Min / Max	22.05/31.34	23.49/28.82	27.94/28.75	22.05/31.34	27.41/29.88

### Primary outcome: safety and tolerability

Safety laboratory evaluations (hematology, biochemistry, coagulation, and urinalysis) were conducted at scheduled visits during the study. All laboratory values remained within acceptable ranges, and no clinically meaningful or treatment-related abnormalities were identified.

Out of 16 participants enrolled in the study, 14 participants (87.5%) reported a total of 56 adverse events (AEs) (Table 2). Thirty-nine AEs were considered by the investigator to be certainly related, and 4 were possibly related to study treatment administration (either the active or placebo). The remaining AEs were considered unlikely to be related (*n* = 3) or unrelated (*n* = 10) to the study treatment. Out of the 56 AEs, 49 were mild, and 7 were moderate in severity. No serious adverse events (SAEs) were reported during the study. In 12 participants (10 from the active treatment group and two from the placebo group), 39 localized injection-site reactions were documented and judged to be certainly related to the study treatment administration procedure. All certainly related AEs were mild in severity. The 4 possibly related AEs included systemic symptoms such as fatigue, myalgia, and chills. All adverse events resolved without sequelae.

The list of unlikely related and unrelated AEs includes abdominal pain, vertigo, delayed recurrence of left arm tenderness, rhinorrhea, viral bronchitis, intermittent myalgia post-viral illness, pneumonia, lethargy, mechanical back pain, epigastric pain, headache due to caffeine cessation, constipation, and fatigue secondary to insomnia. Eight AEs were reported in 3 participants from the placebo group. Of these, 5 were injection-site reactions considered certainly related to placebo administration, while the remaining 3 AEs were assessed as unrelated. A summary of adverse events is presented in Table 2.

**Table 2:** Summary of treatment emergent adverse events by system organ class and preferred term

Treatment group	AV-1959R 100 µg		AV-1959R 300 µg		Pooled		Placebo	
Number of participants	6		6		12		4	
System organ class/preferred term	Events	Participants	Events	Participants	Events	Participants	Events	Participants
	**N**	**N(%)**	**N**	**N(%)**	**N**	**N(%)**	**N**	**N(%)**
**Any TEAE**	**22**	**5 (83.3)**	**26**	**6 (100)**	**48**	**11 (91.7)**	**7**	**3 (75)**
**Ear and labyrinth**	**1**	**1 (16.7)**	**-**	**-**	**1**	**1 (8.3)**	**-**	**-**
Vertigo	1	1 (16.7)	-	-	1	1 (8.3)	-	-
**Gastrointestinal**	**1**	**1 (16.7)**	**1**	**1 (16.7)**	**2**	**2 (16.7)**	**1**	**1 (25)**
Abdominal pain	1	1 (16.7)	-	-	1	1 (8.3)		
Upper Abdominal pain	-	-	-	-	-	-	1	1 (25)
Constipation	-	-	1	1 (16.7)	1	1 (8.3)	-	-
**Infections and infestations**	**2**	**2 (33.3)**	**-**	**-**	**2**	**2 (16.7)**	**-**	**-**
Bronchitis viral	1	1 (16.7)	-	-	1	1 (8.3)	-	-
Pneumonia	1	1 (16.7)			1	1 (8.3)	-	-
**Musculoskeletal and connective tissue**	**1**	**1 (16.7)**	**1**	**1 (16.7)**	**2**	**2 (16.7)**	**-**	**-**
Back pain	-	-	1	1 (16.7)	1	1 (8.3)	-	-
Myalgia	1	1 (16.7)	-	-	1	1 (8.3)	-	-
**Nervous system**	**1**	**1 (16.7)**	**-**	**-**	**1**	**1 (8.3)**	**-**	**-**
Lethargy	1	1 (16.7)	-	-	1	1 (8.3)	-	-
**Respiratory**,** thoracic**,** and mediastinal**	**-**	**-**	**-**	**-**	**-**	**-**	**1**	**1 (25)**
Rhinorrhoea	-	-	-	-	-	-	1	1 (25)
**General disorders and injection site conditions**	**16**	**5 (83.3)**	**24**	**5 (83.3)**	**40**	**10 (83.3)**	**5**	**2 (50)**
Chills	2	1 (16.7)	-	-	2	1 (8.3)	-	-
Discomfort	-	-	1	1 (16.7)	1	1 (8.3)	-	-
Fatigue	1	1 (16.7)	1	1 (16.7)	2	2 (16.7)	-	-
Injection site discomfort	-	-	2	1 (16.7)	2	1 (8.3)	-	-
Injection site erythema	-	-	3	3 (50)	3	3 (25)	-	-
Injection site inflammation	-	-	1	1 (16.7)	1	1 (8.3)	-	-
Injection site pain	11	5 (83.3)	13	4 (66.7)	24	9 (75)	5	2 (50)
Injection site swelling	1	1 (16.7)	2	2 (33.3)	3	3 (25)	-	-
Pain	1	1 (16.7)	-	-	1	1 (8.3)	-	-
Tenderness	-	-	1	1 (16.7)	1	1 (8.3)	-	-

### Secondary outcome: immunogenicity

**Fig. 2:**
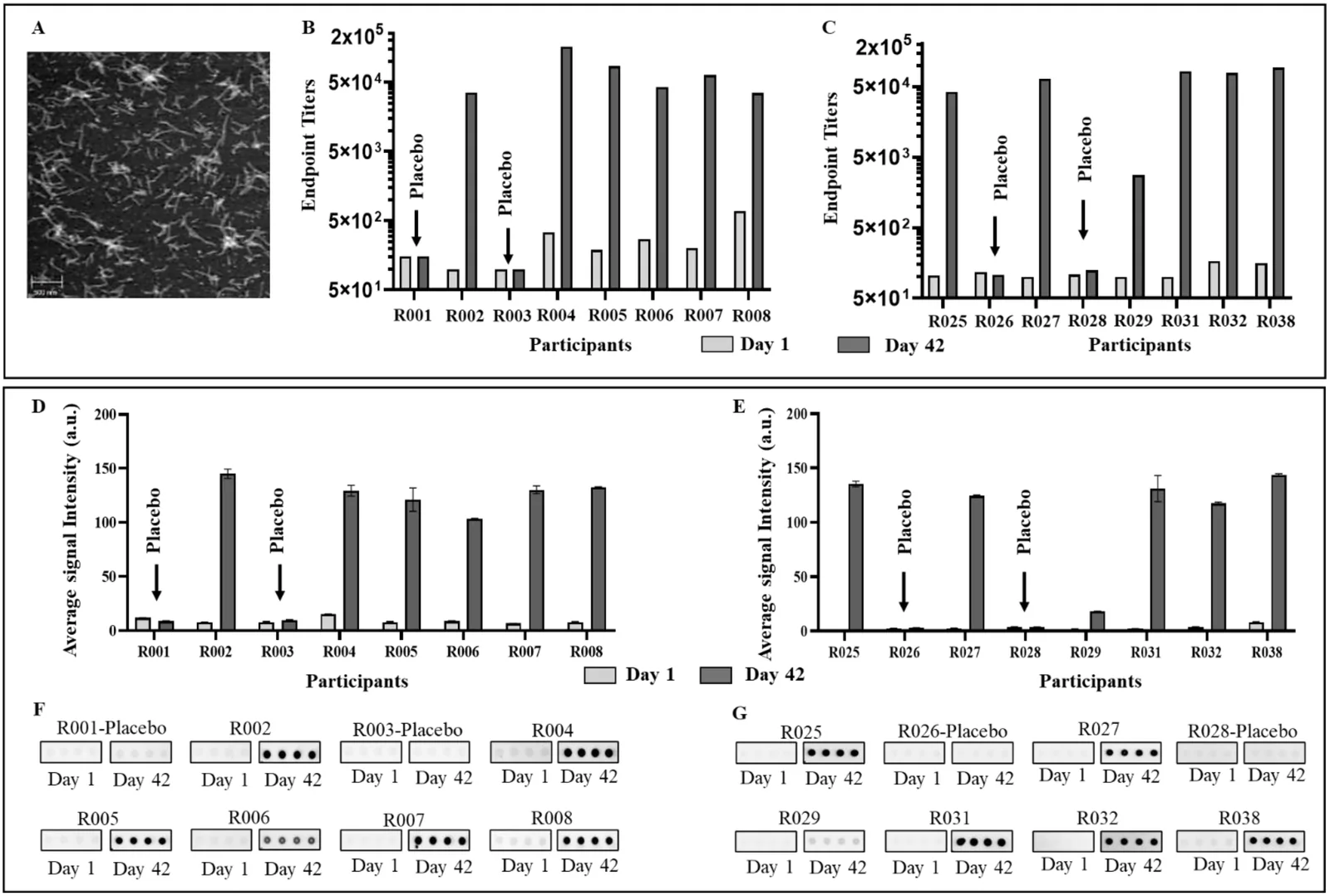
Potent induction of anti-Aβ₄₂ antibodies by AV-1959R in healthy volunteers. (**A**) Atomic force microscopy (AFM) image of the Aβ₄₂ peptide preparation (10 µM) used for ELISA plate coating, showing a heterogeneous mixture of protofibrillar and fibrillar structures. (**B**, **C**) Endpoint anti-Aβ₄₂ IgG titers in participants immunized with 100 µg (**B**) or 300 µg (**C**) of AV-1959R, demonstrating robust antibody induction following two priming doses by Day 42. (**D**, **E**) Quantitative protein-microarray signal intensities (Bars represent mean from 4 wells ± SEM), and (**F**, **G**) representative microarray images showing the binding of individual participants’ sera (1:100 dilution) from Cohort 1 (**D**, **F**) and Cohort 2 (**E**, **G**) to Aβ₄₂ protofibril/fibril (PF/F) antigens identical to those used in the ELISA (10 µM)

To analyze anti-Aβ antibody titers in sera collected from participants in Cohorts 1 and 2, we developed a validated ELISA SOP in which wells of plates were coated with predominantly protofibrillar and fibrillar forms of Aβ_42_ (Fig. 2A). Because antibody responses reached their peak following the second immunization (Day 42), sera collected at this time point were selected for detailed characterization of antibody titers, specificity and functional properties unless otherwise indicated. All six participants who had been primed with a 100 µg dose of AV-1959R mounted robust antibody responses against Aβ_42_ protofibrils and fibrils by Day 42. Endpoint titers (ETs) ranged from 1:35,000 in participant R002 to 1:165,000 in participant R004, with a cohort geometric mean titer (GMT 60,240) (Fig. 2B). In Cohort 2, five of six participants who received the 300 µg dose of AV-1959R also developed strong antibody responses, with a GMT of 69,530 (ET ranging from 1:42,174 to 1:92,632), comparable to the levels observed in the 100 µg group. One participant, R029, showed only a modest response with ET equal to 1:2,780, yet this remained ~ 20-fold above the baseline anti-Aβ_42_ antibody titer (Fig. 2C). Group-level analysis confirmed that both 100 µg and 300 µg doses of AV-1959R were highly immunogenic, with all vaccinated participants eliciting antibodies that specifically recognized pathological Aβ_42_ conformers. 10 of 12 individuals across both cohorts generated consistently high antibody titers (combined GMT 49,490). Notably, one participant (R004) showed an exceptionally strong response, while another (R029) exhibited a minimal response.

By contrast, none of the placebo recipients (*n* = 4) showed measurable responses, and all maintained baseline anti-Aβ_42_ antibody levels (GMT 156; mean ET 187 ± 149), consistent with values observed across participants before vaccination at study entry (Fig. 2B, C, and Supplementary Table 1). Quantitative comparison revealed no significant difference (*p* = 0.7367) between the groups receiving the 100 µg and 300 µg priming regimens, indicating that dose escalation beyond 100 µg does not provide additional immunogenic benefit.

Given the inherent aggregation-prone nature of amyloid peptides, we recognize that the use of bulk polystyrene immunoassay plates for coating Aβ_42_ protofibrils and fibrils may significantly influence their conformation and structural integrity [34, 35]. To mitigate potential artifacts arising from peptide-surface interactions, we evaluated antibody binding using a nitrocellulose-based microarray assay. As shown in Fig. 2D-G, sera collected from all participants after priming immunizations effectively recognized Aβ_42_ protofibrils and fibrils on the nitrocellulose membrane. Antibody endpoint titers correlated with microarray binding intensity across both cohorts: Pearson *r* = 0.7354, *p* = 0.0012. 

**Fig. 3:**
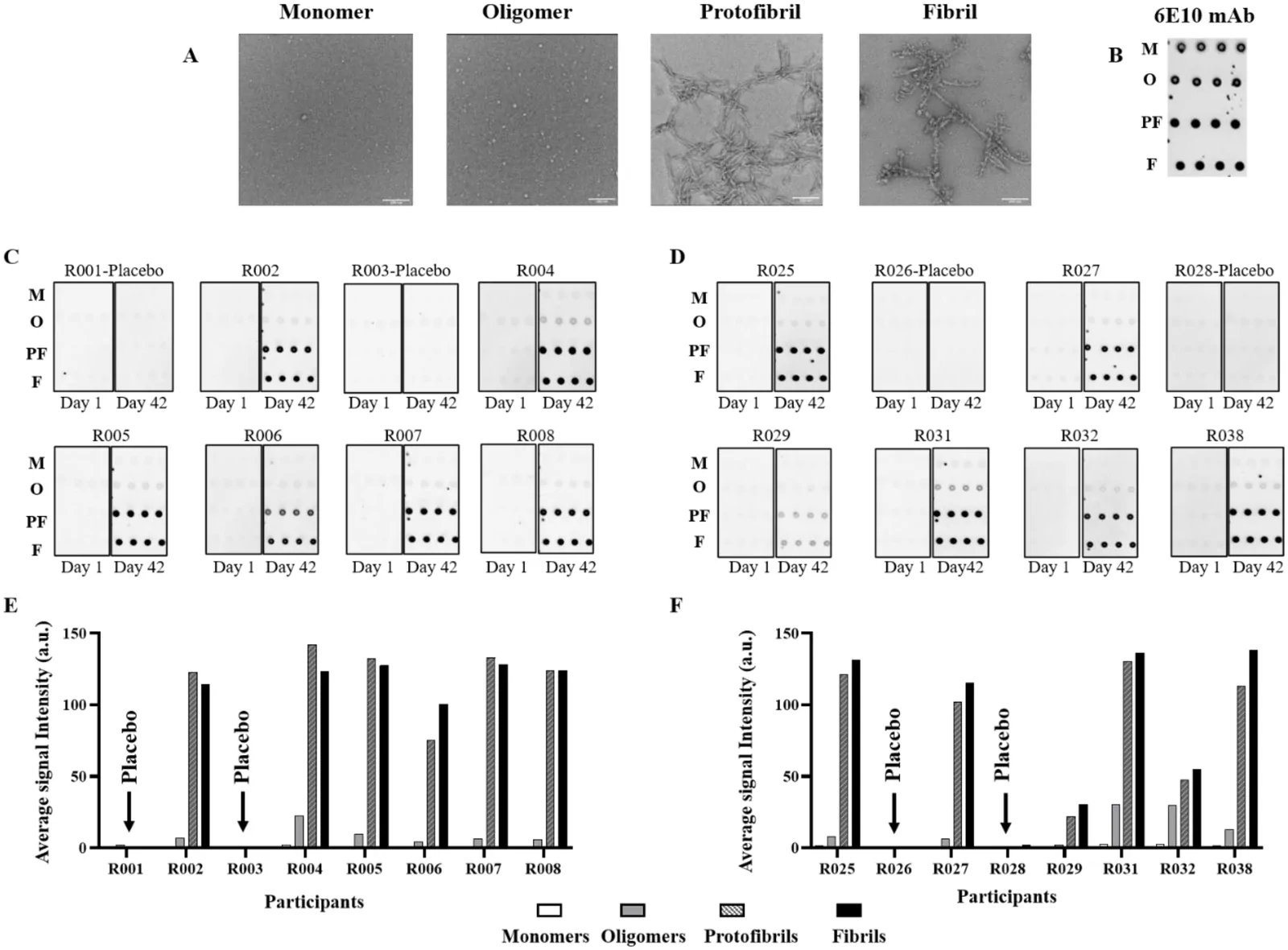
Vaccine-induced antibodies preferentially recognize pathological Aβ₄₂ conformers (**A**) Electron Microscopy (EM) images of different species of Aβ_42_ peptides spotted onto nitrocellulose microarray slides. Scale bar, 100 nm. (**B**) Microarrays probed with 6E10 mAb to confirm equal spotting of all Aβ conformers. (**C**-**F**) Protein microarray dot-blot analysis of sera (1:100 dilution) from participants in Cohort 1 (**C**) and Cohort 2 (**D**), demonstrating that vaccine-induced antibodies preferentially bind aggregated Aβ_42_ species (protofibrils and fibrils) rather than monomeric or oligomeric forms, indicating selective recognition of pathological conformations. Each Aβ_42_ conformation was spotted in quadruplicate. Average signal intensity is shown for Cohort 1 (**E**) and Cohort 2 (**F**)

Modern refinements of the ‘amyloid cascade hypothesis’ [36, 37] emphasize that Aβ oligomer formation is the critical initiating event for downstream pathological processes, including amyloid fibrilization, Aβ_42_ plaque formation, accumulation of tau pathology, inflammation, oxidative stress, and synaptic and neuronal loss [4–6, 9, 10, 38–45]. The Aβ_42_ peptide is intrinsically disordered, lacks a stable tertiary structure, and readily self-associates in aqueous buffers [46]. To profile the specificity of AV-1959R-induced antibodies across Aβ_42_ conformational states, we printed monomeric, oligomeric, protofibrillar, and fibrillar Aβ_42_, prepared according to defined protocols [30, 47], onto nitrocellulose microarrays and probed them with sera from individual trial participants. The morphology and sample homogeneity of each preparation were analyzed by electron microscopy (EM) (Fig. 3A) and confirmed by AFM immediately before and after spotting (Supplementary Fig. 1). As expected, positive control mAb 6E10, which recognizes the N-terminal region of Aβ, bound to monomeric Aβ_42_ as well as all aggregated Aβ_42_ conformations, thereby validating antigen presentation in the microarray assay (Fig. 3B). Sera collected on Day 42 from vaccinated participants in Cohorts 1 and 2 showed strong binding to Aβ_42_ protofibrils and fibrils, weaker reactivity toward oligomers, and minimal or no binding to monomeric Aβ_42_ (Fig. 3D-F). To confirm specificity, we demonstrated that baseline sera (Day 1) from the vaccinated participants showed no detectable binding to any Aβ_42_ conformer. As we expected and consistent with ELISA data, sera from placebo recipients at both Day 1 and Day 42 showed no evident binding to any morphological forms of Aβ_42_ (Fig. 3D-F).

This preferential recognition of aggregated Aβ species was further confirmed by a competition ELISA. Pre-incubation of pooled sera with aggregated Aβ₄₂ (2.5 µM) reduced binding to protofibrils and fibrils by up to 96%, whereas much higher concentrations (150–200 µM) of the monomeric Aβ_1-11_ peptide were required for complete inhibition. The estimated IC₅₀ values were 16.9 µM for Aβ_1-11_ and 0.01 µM for the soluble Aβ₄₂ protofibril/fibril mixture (Supplementary Fig. 2), consistent with markedly higher apparent affinity for aggregated Aβ species.

In addition, to directly characterize vaccine-induced Aβ-specific antibodies independent of serum background components, anti-Aβ antibodies were affinity-purified from pooled Day 42 sera of AV-1959R-vaccinated participants (Cohorts 1 and 2). The purified antibody fraction was quantified, and binding to a mixture of Aβ_42_ protofibrils and fibrils was assessed by ELISA (Supplementary Fig. 3). The resulting concentration-response curve demonstrated saturable, dose-dependent binding, confirming preserved functional activity and high-affinity recognition of aggregated Aβ species following purification. Notably, we demonstrated that, unlike lecanemab, purified AV-1959R-induced antibodies recognized the pyroglutamate-modified Aβ_3-11_ (pE_3_Aβ_11_) peptide (Supplementary Fig. 4).

Collectively, these findings demonstrate that AV-1959R-induced polyclonal antibodies preferentially and robustly recognize aggregated Aβ_42_ species (protofibrils and fibrils), while exhibiting markedly lower reactivity toward soluble low-order oligomeric forms and pyroglutamate-modified Aβ_3-11_ and minimal to no binding to monomeric Aβ_42_.

### Quantitative analysis of the dynamics of antibody production after priming and a single booster

**Fig. 4:**
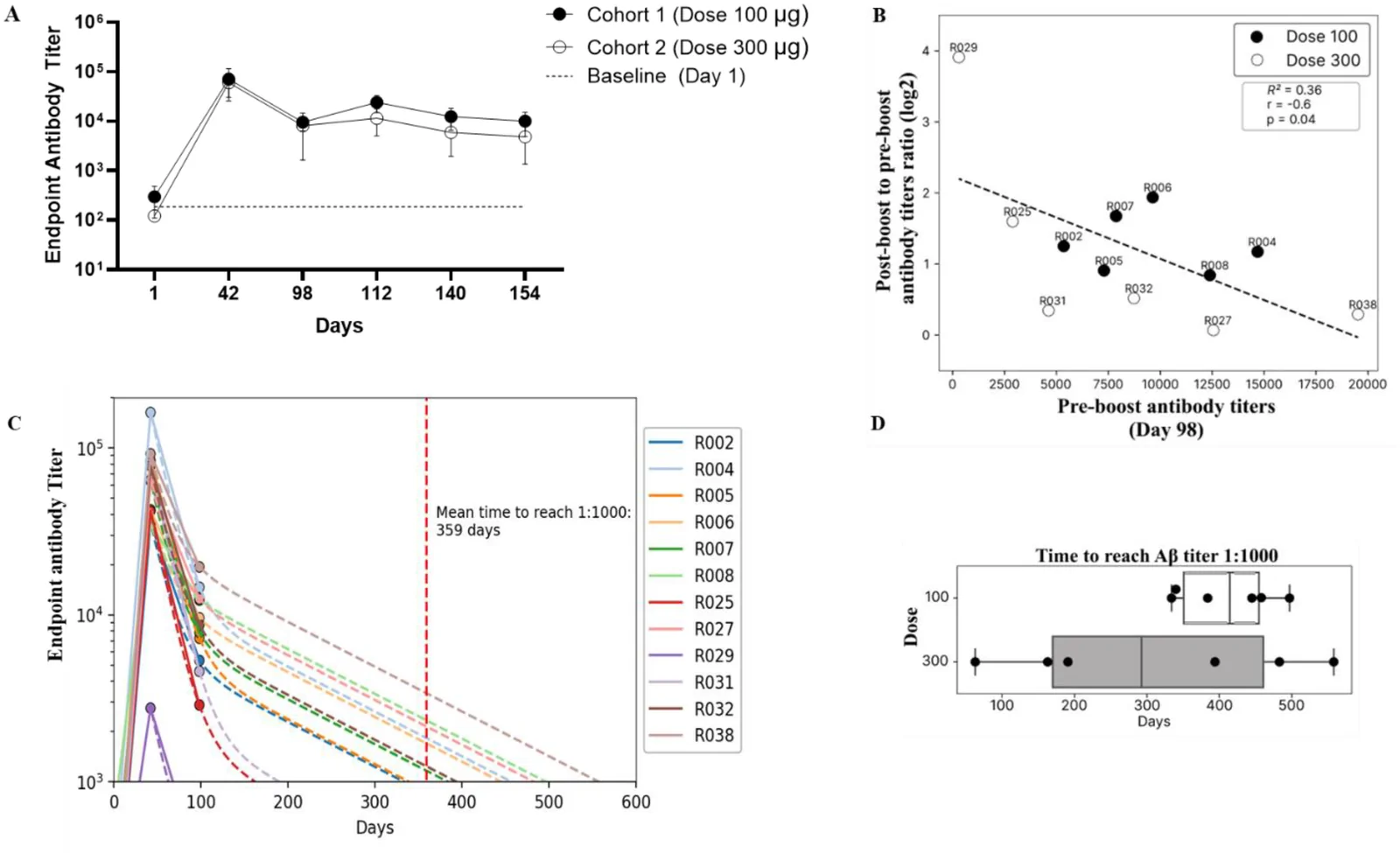
Quantitative analysis of serum antibody dynamics following priming and booster immunization with AV-1959R. (**A**) Mean Endpoint anti-Aβ₄₂ IgG titers measured at multiple time points (Day 0 to Day 154) for participants receiving 100 µg and 300 µg doses of AV-1959R. Antibody titers are plotted on a logarithmic scale. Data are presented as mean ± SD. (**B**) Correlation between the log2 ratio of post-boost/pre-boost titers and pre-boost antibody levels at Day 98. Linear regression is shown as a dashed line. Pearson’s correlation coefficient (r), *p*-value, and coefficient of determination (R²) are indicated on the plot. Participants receiving 100 µg are shown as filled circles; those receiving 300 µg are shown as open circles. (**C**-**D**) Model-estimated time required for antibody titers to decline to 1:1000 after the priming immunizations. Predicted antibody dynamics are shown as dashed lines, with the mean time to reach a titer of 1:1000 indicated by the red vertical line. No significant difference was observed between the 100 µg and 300 µg dose groups

To be effective, recombinant protein-based vaccines should elicit a robust immune response after just two doses, and the data above demonstrate that AV-1959R achieved this critical goal. The next requirement for an effective vaccine is the induction of long-lasting immune responses. Per study design, participants in Cohorts 1 and 2 were boosted on Day 98, eight weeks after priming immunizations (Fig. 1B). Quantitative analysis of antibody kinetics allowed direct comparison between dose groups, correlation of priming and boosting responses, and estimation of the optimal priming-boosting interval (Fig. 4). After boosting on Day 98, antibody titers peaked at Day 112 (Fig. 4A), and the magnitude of the booster response was inversely correlated with the Day 98 pre-boost levels (Fig. 4B). Thus, individuals with lower antibody levels before boosting exhibited greater fold-increases following the booster. Short-term immunity is driven by rapidly produced antibodies that decline quickly, while long-term immunity relies on a small, stable population of long-lived plasma cells that reside in bone marrow and continuously produce antibodies for years [48, 49]. To assess antibody durability, we applied mathematical modeling to separate the response into short-term and long-term components (see Methods, Model for antibody kinetics). The fitted bi-exponential model yielded rate constants of k₁ = 0.059 day⁻¹ and k₂ = 0.0062 day⁻¹, corresponding to half-lives of approximately 12 and 112 days for the short-term and long-term antibody titers, respectively. These values indicate a rapid early decline followed by a more durable antibody fraction, consistent with the activity of long-lived plasma B cells after boosting. After booster immunization, sustained antibody levels were primarily driven by expansion of the long-lived B cells. We further assessed durability by estimating the time required for endpoint titers to decline to 1:1000 after priming, a level approximately 5-fold higher than the mean baseline titer across all participants. Model projections indicated a mean persistence of 359 days across both dose groups, with no significant difference between 100 µg and 300 µg cohorts (Fig. 4C, D). Taken together, these data suggest that a boosting interval of approximately one year would be optimal for sustaining long-term antibody levels above protective thresholds.

### AV-1959R vaccination elicits IgG antibodies that bind Aβ plaques in transgenic AD mice and the human cerebral cortex

**Fig. 5:**
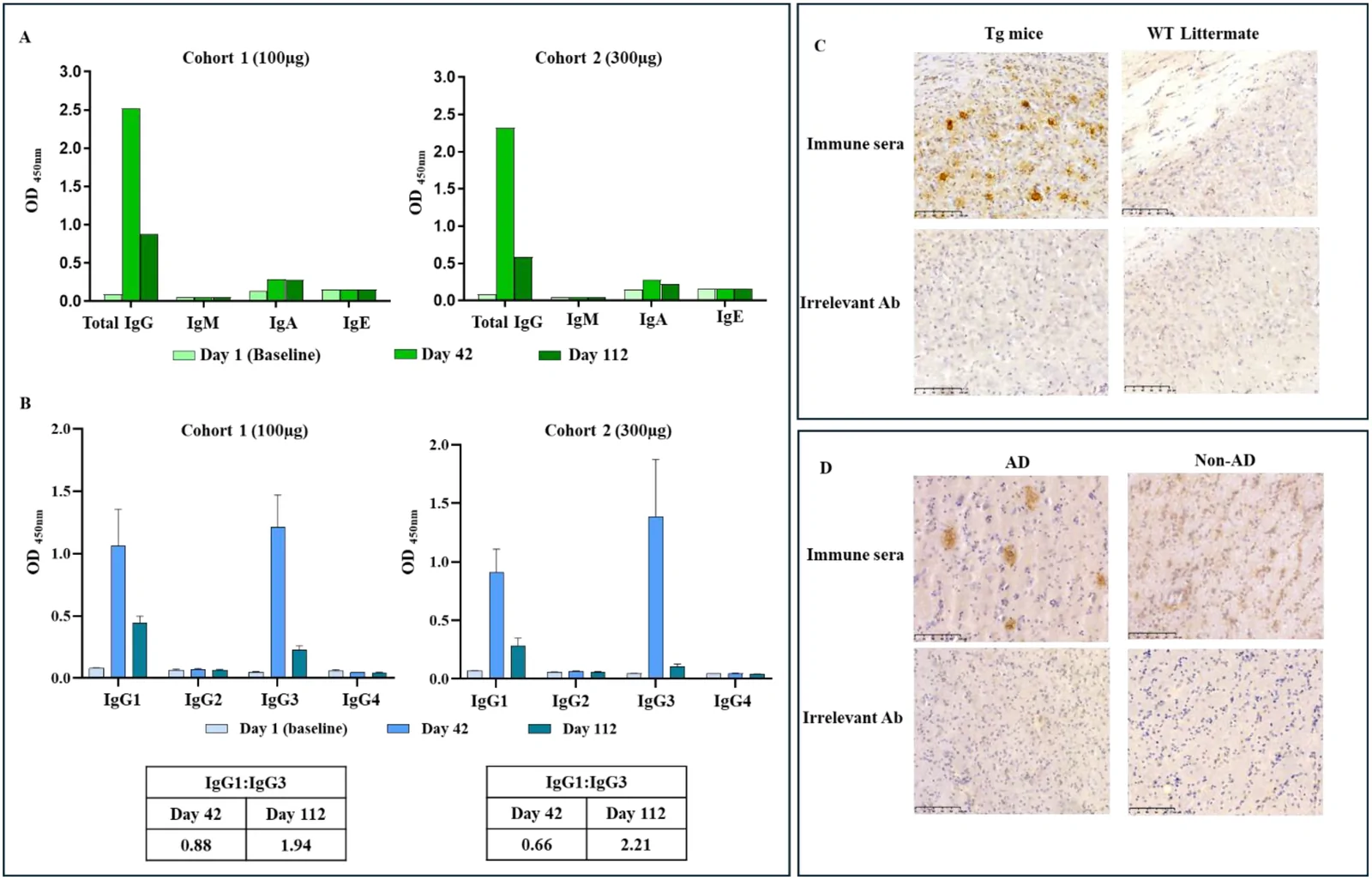
AV-1959R induces predominantly IgG1 and IgG3 antibody subclasses that specifically recognize amyloid plaques. (**A**) AV-1959R elicited strong total IgG antibody responses, comprising mainly the (**B**) IgG1 and IgG3 subclasses. Antibody isotypes were evaluated in pooled serum samples collected at baseline (Day 1), post-priming (Day 42), and post-booster (Day 112) at a 1:3,200 dilution. IgG subclasses were analyzed in pooled sera at a 1:200 dilution. Bars represent mean ± SEM. (**C**,** D**) Responder sera bound to amyloid plaques in brain tissue from an AD mouse model but not from wild-type littermates (**C**) and recognized amyloid plaques in human AD brain sections but not in non-AD controls (**D**)

Previously, we showed that a MultiTEP-based Aβ vaccine elicits cellular immune responses to MultiTEP epitopes without triggering autoreactive T helper (Th) cells against pathological amyloid [24]. In this Phase 1 trial, PBMCs were not collected, and Th cell responses were not analyzed directly. However, it is well established that T memory cells, particularly follicular helper T cells (Tfh), are critical for the generation and persistence of IgG antibodies following immunization [50, 51]. Accordingly, we analyzed antibody isotypes in pooled sera from participants vaccinated with 100–300 µg of AV-1959R. Elevated IgG antibodies specific to Aβ_42_ protofibrils and fibrils were observed after both priming (Day 42) and boosting (Day 112) (Fig. 5A). The selective induction of IgG antibodies against aggregated pathological Aβ species strongly suggests effective Tfh-mediated germinal center B-cell responses, consistent with the mechanism of T-dependent protein vaccines [52, 53]. Subclass analyses of individual sera confirmed that vaccination induced only IgG1 and IgG3, independent of dose or timepoint (priming vs. boosting). After priming, the IgG1:IgG3 ratio was 0.88 in the 100 µg and 0.66 in the 300 µg groups, whereas after boosting, IgG1 predominated (IgG1:IgG3 = 1.94 for 100 µg and 2.21 for 300 µg) (Fig. 5B). This shift indicates a durable, Th-dependent, class-switched IgG response.

Finally, to assess the biological relevance of vaccine-induced antibodies, we examined their ability to recognize amyloid pathology in brain tissues. Immune sera from vaccinated participants bound specifically to Aβ plaques in the brains of Tg AD mouse models, but not in non-transgenic littermate controls (Fig. 5C). Importantly, the same immune sera also recognized plaques in cortical sections from human AD cases, while no staining was observed in non-AD controls (Fig. 5D). These immunohistochemistry findings confirm that AV-1959R induces functionally relevant IgG antibodies that selectively target both soluble pathological forms of Aβ_42_ peptides (Fig. 2, 3) and amyloid plaques (Fig. 5D) - a key hallmark of Alzheimer’s disease that accumulates years before tau pathology and clinical symptom onset [10]. Taken together with data presented in Fig. 2–5, these results demonstrate that AV-1959R elicits strong, isotype- and subclass-specific IgG responses that are mechanistically grounded and biologically relevant to human AD pathology [54].

## Discussion

The landscape of Aβ-targeted immunotherapies has evolved considerably, shifting from a treatment focus to a proactive, preventive approach [55–57]. While mAbs have demonstrated modest clinical benefits in early AD [58], their complexity, cost, safety risks (e.g., ARIA), and requirement for repeated infusions or subcutaneous injections make them impractical for widespread preventive use in cognitively normal preclinical Aβ-positive individuals [59, 60]. In contrast, a safe and durable Aβ vaccine capable of sustaining immunity with a single annual booster could offer a feasible, cost-effective, and globally scalable solution for AD prevention among the estimated 315 million individuals at the preclinical AD stage worldwide [61, 62].

The data from the AN1792 trials are the most comprehensively analyzed and reported among all active Aβ vaccine trials to date [63–80]. In this first active Aβ immunotherapy trial, approximately 20% of treated AD patients mounted anti-Aβ_42_ antibody responses with pre-defined endpoint titers ≥ 1:2200 [68]. Regrettably, a larger Phase 2a trial involving 372 patients with mild to moderate AD was halted after approximately 6% of immunized participants developed subacute neurological deterioration accompanied by cerebrospinal fluid lymphocytosis and variable focal white matter abnormalities on MRI [81]. This adverse event was diagnosed as aseptic meningoencephalitis and was associated with perivascular infiltrates thought to contain autoreactive Aβ-specific T cells [65, 66]. Although the MRI findings observed after AN1792 have retrospectively been recognized as being consistent with ARIA-E, the underlying mechanisms are thought to differ. AN1792-associated meningoencephalitis is believed to have resulted primarily from autoreactive Aβ-specific T-cell responses, whereas ARIA associated with anti-Aβ monoclonal antibodies is thought to arise predominantly from antibody-mediated vascular amyloid clearance [82]. Consistent with this distinction, the AN1792 trials did not report ARIA-H or superficial siderosis, although postmortem analyses of some immunized patients demonstrated increased cortical Perls’ Prussian Blue-positive microhemorrhages, the histopathological correlate of MRI-detected cerebral microhemorrhages [78].

Importantly, a long-term clinical and neuropathological follow-up of AD patients who received the first-generation, modestly immunogenic AN-1792 vaccine demonstrated evidence of amyloid plaque removal up to 14 years after the final immunization in individuals who had mounted an antibody response. A significant inverse correlation was observed between post-vaccination peripheral anti-Aβ antibody titers and postmortem amyloid plaque burden. However, despite these histopathological improvements, AN-1792 vaccination did not halt or prevent clinical disease progression or the accumulation of neurofibrillary tau tangles [80].

Almost 23 years ago, our team proposed that the weak immune responses seen in vaccinated AD patients were probably due to tolerance to fibrillar Aβ_42_, age-related immunosenescence, and the adjuvant [83, 84]. To address these issues, we created a series of epitope vaccines in which self-Aβ, tau, or α-synuclein B cell epitopes are fused to foreign T helper epitopes capable of activating human Th cells (the MultiTEP platform) [20, 21, 26, 28, 29, 85, 86]. AV-1959R is the latest version of this platform and differs significantly from AN-1792 and other clinically tested active vaccines. It was designed to (i) overcome self-tolerance by activating Th cells specific for foreign epitopes in MultiTEP; (ii) provide broad coverage across populations despite extensive HLA class II diversity; (iii) counteract immunosenescence by recruiting both naive and memory Th cells specific for common pathogens and vaccine antigens; and (iv) prevent induction of autoreactive Aβ-specific Th cells involved in the AN1792-associated meningoencephalitis. Consistent with this design and with preclinical studies demonstrating efficacy, safety, and immunogenicity of AV-1959D and AV-1959R [21, 24, 25, 27, 87], the current phase 1 trial shows that AV-1959R elicits strong antibody responses without MRI evidence of ARIA-E or ARIA-H in vaccinated participants without clinically apparent cognitive impairment. However, due to the small sample size and the lack of cerebral amyloid pathology in this group, definitive safety conclusions cannot be drawn. Therefore, a larger Phase 2 trial (IND 30925-0004; IRB-Pro00095455) is underway, which will monitor ARIA-E and ARIA-H in cognitively unimpaired participants with biomarker-confirmed preclinical AD across a range of amyloid levels (low, < 40CL, and high, > 40CL). Demonstrating safety and target engagement in this population will enable subsequent evaluation of biological efficacy using established AD biomarkers and support the further development of AV-1959R as a preventive immunotherapy for AD in non-cognitively impaired individuals, consistent with recently proposed amyloid-targeting strategies for disease prevention [88].

Here, we report the first evaluation of AV-1959R in healthy volunteers (mean age 51.6, range 43–60 years). The vaccine was safe and well-tolerated; no serious adverse events (SAE) or treatment-emergent adverse events leading to withdrawal were observed in the 12 vaccine recipients. Reported AEs were limited to mild, transient injection-site reactions, occurring in 10 of 12 vaccine recipients and 2 of 4 placebo recipients, all resolving spontaneously. 

Importantly, no ARIA has been observed in the 12 vaccinated non-cognitively impaired participants, and our phase 2 trial will provide more precise estimates of safety in preclinical AD participants with defined amyloid plaque burden.

Immunogenicity analyses revealed that 11 of 12 participants receiving either 100–300 µg doses of AV-1959R developed robust anti-Aβ IgG responses, reaching exceptionally high antibody levels. The single remaining participant, although showing a comparatively weaker response after priming, still achieved antibody titers more than 20-fold higher than their own baseline. Importantly, after administering a booster dose, antibody titers nearly doubled, reaching ET 1:4529.

Functional characterization of immune sera revealed strong binding to Aβ_42_ fibrils and protofibrils, moderate recognition of oligomers, and minimal to negligible binding to monomeric Aβ_42_ peptide (Fig. 2, 3). Accordingly, antibodies induced by AV-1959R not only recognized amyloid plaques in brain sections from AD cases (Fig. 5) but also preferentially bound aggregated rather than monomeric Aβ_42_ peptide. Although protofibrils are operationally defined as Aβ aggregates generated during in vitro fibrillization, and their existence as distinct native species in the human brain remains uncertain, they have been widely used as experimental targets for the development and clinical evaluation of anti-Aβ antibodies, including lecanemab. Therefore, inclusion of protofibrils facilitates comparison of AV-1959R-induced antibodies with existing anti-Aβ immunotherapies. Although the mechanism underlying this binding profile was not directly investigated in the present study, several features of AV-1959R may contribute to it. First, the vaccine presents three copies of the Aβ_1–11_ B-cell epitope in a multivalent configuration. Second, characterization of the cGMP drug product by dynamic light scattering demonstrated that recombinant AV-1959R forms nanoparticle-like assemblies, and under native conditions, the protein further self-associates into higher-molecular-weight oligomeric species (~ 300 ± 200 kDa) (Supplementary Fig. 5). These structural features likely promote repetitive epitope display, which is known to enhance B-cell receptor crosslinking and preferentially stimulate B cells recognizing repetitive antigen arrays. Such antibodies would be expected to bind aggregated Aβ species more efficiently than monomeric Aβ. Consistent with this interpretation, vaccine-induced antibodies exhibited substantially stronger binding to fibrillar and protofibrillar Aβ than to monomeric peptide. Interestingly, antibodies induced by AN1792 were also reported to recognize the free N-terminus of Aβ, with the dominant epitope mapping to Aβ_1–10_, and plaque binding was abolished by preabsorption with N-terminal Aβ peptides, demonstrating that antibodies directed against linear N-terminal epitopes can selectively recognize aggregated Aβ in brain tissue [63, 89].

Notably, antibodies generated in participants immunized with AV-1959R also bound pyroglutamate-modified Aβ_3-11_ peptide (Supplementary Fig. 4). Because this peptide contains the N-terminal epitope present in full-length pE_3_Aβ_42_, these data suggest the potential for recognition of pE_3_Aβ_42_, a highly aggregation-prone species implicated in early amyloid seeding.

This selective binding profile is significant, as it may improve the therapeutic index by avoiding competition from abundant, non-pathogenic monomeric Aβ and by lowering the risk of interfering with potential physiological functions of soluble monomeric Aβ_42_ at neuronal synapses, while still enabling targeting of clinically relevant aggregated and modified Aβ species [90, 91].

Quantitative analyses revealed no statistically significant differences in antibody titers between participants immunized with 100 µg and 300 µg doses of the vaccine, indicating that higher antigen doses do not necessarily enhance immunogenicity. Furthermore, antibody kinetics analyses showed a trend toward faster titer decline in individuals who entered the booster phase with elevated baseline levels, suggesting that excessive antigen exposure in the presence of pre-existing antibodies may induce feedback inhibition of B-cell activation. To determine optimal booster regimen, mathematical modeling of antibody kinetics was performed, predicting that serum antibody levels in both cohorts would decline to an endpoint titer of approximately 1:1,000 - about fivefold above the mean baseline across all participants - by around 359 days after priming. Accordingly, a Phase 2 trial schedule employing a 100 µg initial dose, a priming dose at week 4, and annual boosters is proposed as a potentially optimal strategy for cognitively normal individuals with blood- and brain-based biomarker evidence of Aβ pathology.

A key immunological feature of AV-1959R is the selective induction of IgG1 and IgG3 subclasses, both potent activators of complement and Fcγ receptor-mediated effector mechanisms, including microglial phagocytosis that promote clearance of aggregated Aβ_42_ species from the brain [50, 51]. This response profile may be advantageous for preventive immunotherapy, in which intervention is initiated before substantial Aβ and tau pathology is established and before neurodegeneration becomes clinically apparent. The absence of IgG2 and IgG4 further strengthens this profile, since IgG4 is associated with immune tolerance and reduced clearance efficiency, whereas IgG2 antibodies are typically directed against carbohydrate antigens and are of limited relevance to amyloid pathology [92]. Additionally, data showed that these antibodies bind strongly to Aβ_42_ fibrils and protofibrils (Fig. 3), as well as to Aβ plaques in the brains of APP/Tg mice and in human AD cases (Fig. 5C, D). Together, these findings underscore that AV-1959R not only elicits high-quality antibody responses but also engages effector pathways ideally suited for the prophylactic prevention of Alzheimer’s.

Only two other Aβ vaccines targeting the N-terminal region of pathological amyloid are currently in clinical development: ACI-24 and UB-311. Patients with mild AD who received UB-311 generated low-level anti-Aβ antibody responses (mean endpoint titer range: 1.8–3.7 log10) [93, 94], and only 33% of adults (25–45 years) with Down syndrome receiving ACI-24 produced low antibody titers [95]. In addition to these low response rates, both vaccines required five to seven administrations over a 12–15-month period. In contrast, the AV-1959R vaccine induced high titers of therapeutically relevant anti-Aβ IgG antibodies in healthy volunteers after only two immunizations, suggesting that it may offer a viable preventive strategy to delay or even halt disease onset in Aβ biomarker-positive but cognitively intact individuals. The consistent 100% responder rate underscores the exceptional immunogenicity of the MultiTEP platform incorporated in AV-1959R with Advax-CpG55.2 adjuvant, distinguishing it from prior Aβ vaccine approaches evaluated in clinical trials [68, 93–100]. The MultiTEP platform incorporates a panel of promiscuous foreign T helper (Th) cell epitopes derived from common pathogens, together with the synthetic PADRE epitope, designed to (i) overcome self-tolerance, (ii) activate both naïve and pre-existing memory Th cells primed by prior infections or vaccinations, (iii) avoid the induction of autoreactive T cells specific to AD-related proteins, and (iv) ensure broad coverage across MHC class II polymorphisms in humans. Extensive preclinical studies have consistently validated these mechanisms [20, 24, 26, 28, 29, 85, 101]. The vaccine is formulated with the Advax-CpG55.2 adjuvant, composed of delta inulin polysaccharide particles and a CpG55.2 oligonucleotide. Delta inulin engages DC-SIGN, a C-type lectin receptor expressed on dendritic cells [102], while CpG55.2 activates toll-like receptor 9 [103]. Together, these components enhance the immunogenicity of the MultiTEP-based vaccine by promoting efficient dendritic cell activation and T-helper cell priming, thereby amplifying the vaccine’s intrinsic capacity to induce potent T-dependent B-cell responses. Advax-CpG55.2 has a well-established safety record in clinical use, with over eight million human doses given as part of the licensed SpikoGen COVID-19 vaccine [104], supporting its potential for preventive vaccination in individuals at the preclinical stage of Alzheimer’s.

Our goal was to develop an Aβ vaccine capable of eliciting strong and durable immune responses in cognitively normal individuals at risk for AD. Recent advances in biomarker research allow for the identification of such preclinical AD individuals with evidence of amyloid pathology, potentially decades before clinical symptoms emerge [105, 106].

## Conclusion

In sum, these Phase 1 results support advancing AV-1959R to an accelerated Phase 2/3 secondary prevention trial in cognitively unimpaired (N-), but Aβ-positive (A+) individuals, regardless of tau status (T − and T+), aged 55 years and older. Importantly, based on our preclinical findings [20, 21, 24, 25, 27, 87] and the clinical results reported here, the protocol for a Phase 2 secondary prevention trial of AV-1959R (IRB-Pro00095455; IND 30925-0004) has been approved for cognitively unimpaired individuals with evidence of Aβ pathology. It incorporates surrogate brain- and blood-based biomarkers, combined with next-generation sequencing (NGS) of PBMCs, to comprehensively profile vaccine-induced immune signatures, including B-cell receptor (BCR) and T-cell receptor (TCR) repertoires and clonal lineages before and after immunization. Moreover, the favorable safety and immunogenicity profile of AV-1959R highlights its potential utility for the future primary prevention in cognitively normal, biomarker-negative (A−/T−/N−) individuals who are at elevated risk of AD due to genetic factors (APP, PSEN1, PSEN2, APOE4, or TREM2 R47H variants) or modifiable clinical conditions such as hypertension, diabetes, and obesity.

## Supplementary Information

Below is the link to the electronic supplementary material.

Supplementary Material 1

## Data Availability

Anonymized individual participant data (IPD) from completed studies, together with supporting clinical trial documents (including the study protocol, statistical analysis plan, and informed consent form), are available from the corresponding author upon reasonable request (M.G.A.; magadjanyan@immed.org). Data will be shared following review and approval of a formal request and completion of a data-sharing agreement, in accordance with institutional policies and applicable ethical and regulatory requirements. Trial registration information is publicly available through the Australian New Zealand Clinical Trials Registry (ACTRN12624000737538) and ClinicalTrials.gov (NCT06831812).
