# Supplementary material for: A randomized, placebo-controlled, double blind Phase 1 trial of the Aβ vaccine, AV-1959R, in non-cognitively impaired participants: prelude for secondary preventive therapy in preclinical AD
Source: Mol Neurodegener. 2026 Jul 22;21:72. doi: 10.1186/s13024-026-00980-y (PMC13746423; doi:10.1186/s13024-026-00980-y)
Supplement: Supplementary file 1 [file 13024_2026_980_MOESM1_ESM.pdf]

Supplementary Figures and Tables

Supplementary Table 1: Endpoint titers of anti-Aβ_{42} antibodies in AV-1959R- and placebo-treated participants.

Rand #	Day 1	Day 42	Day 98	Day 112	Day 140	Day 154
**Cohort 2 – 100 µg**						
**R001 (Placebo)**	154	153	145	126	100	100
**R002**	100	35 625	5 354	12 757	5 333	4 480
**R003 (Placebo)**	100	100	100	100	100	100
**R005**	190	85 235	7 296	13 693	6 694	5 911
**R006**	269	42 728	9 634	36 951	19 911	16 951
**R004**	338	164 643	14 672	33 089	9 827	6 050
**R007**	203	63 707	7 855	25 112	12 628	10 176
**R008**	686	35 115	12 389	22 239	19 968	16 618
**Cohort 2 – 300 µg**						
**R025**	105	42 174	2 894	8 770	3 237	2 786
**R026 (Placebo)**	118	107	122	100	100	122
**R027**	100	64 924	12 558	13 169	9 115	6 556
**R028 (Placebo)**	109	124	135	117	108	281
**R029**	100	2 780	301	4 529	1 101	1 125
**R038**	157	92 632	19 520	23 885	12 467	11 359
**R031**	100	81 848	4 620	5 870	2 814	1 849
**R032**	167	78 281	8 733	12 513	6 769	5 278

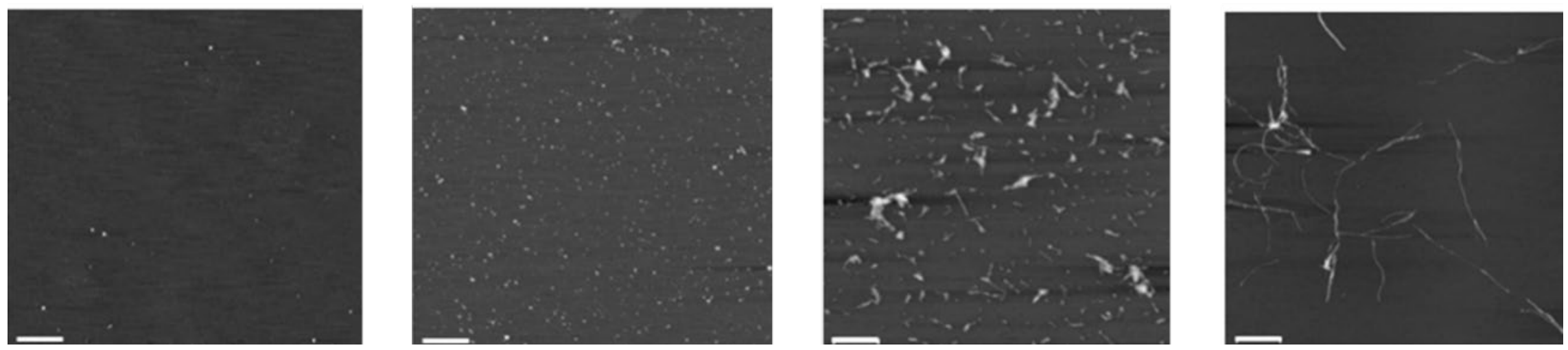

Supplementary Figure 1. Atomic Force Microscopy (AFM) images of Aβ_{42} peptide conformers spotted onto microarray slides. scale bars are 500 nm.

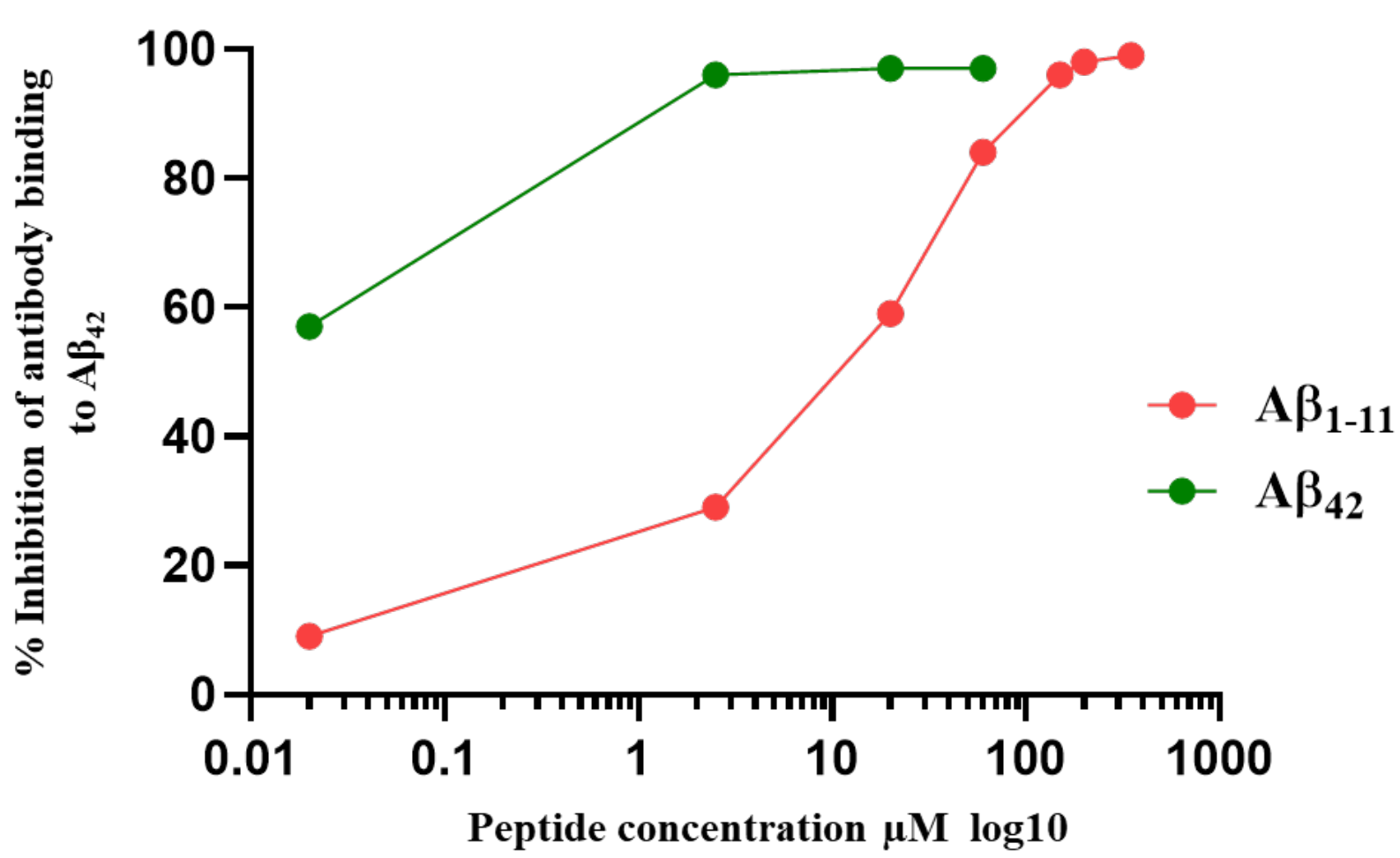

Supplementary Figure 2. Competition ELISA demonstrating the specificity of vaccine-induced antibodies. Pre-incubation of pooled AV-1959R immune serum with soluble Aβ_{42} peptide (2.5 µM) containing a protofibril/fibril mixture almost completely blocked binding to plate-bound Aβ_{42}, whereas approximately 150-200 µM of Aβ_{1-11} peptide was required to achieve comparable inhibition. IC_{50} is 16.9 µM for Aβ_{1-11} and 0.01 µM for the soluble Aβ_{42} protofibril/fibril mixture.

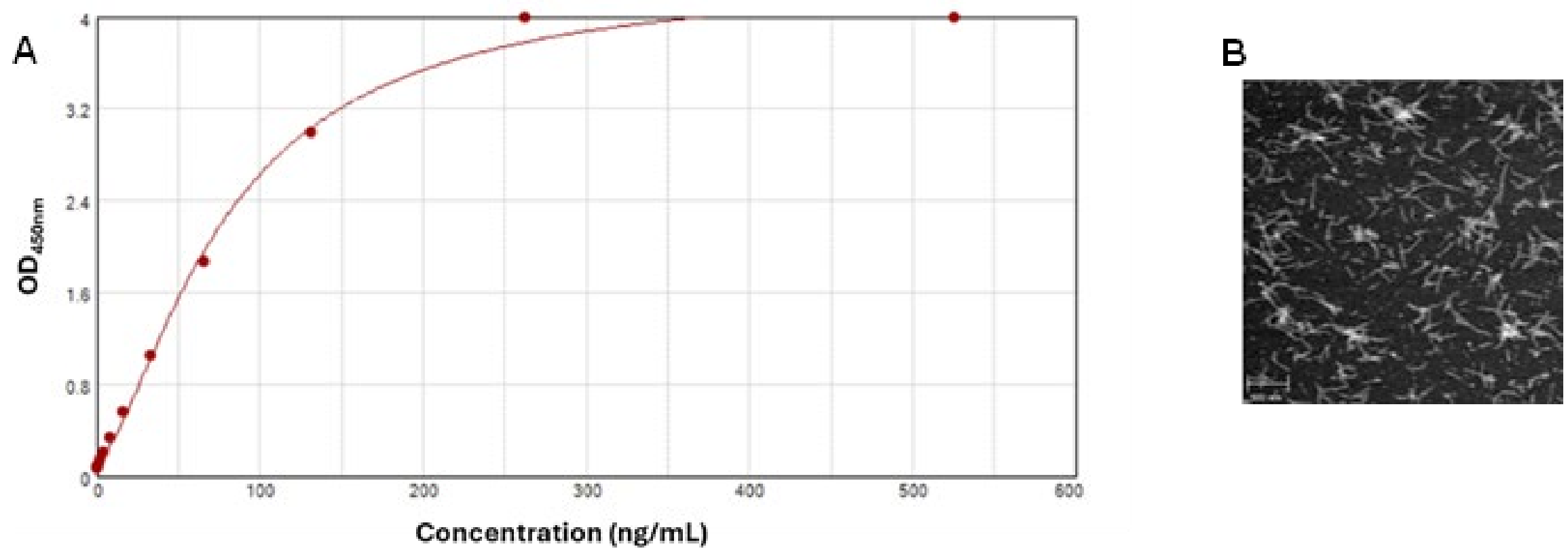

Supplementary Figure 3. Affinity purification and concentration-dependent binding of AV-1959R–induced anti-Aβ antibodies with $A\beta_{42}$ protofibrils/fibrils. Aβ-specific antibodies were affinity-purified from pooled Day 42 immune sera of vaccinated participants. (A) Antibody concentration was determined, and binding to a mixture of $A\beta_{42}$ protofibrils and fibrils was evaluated by ELISA. (B) Atomic force microscopy (AFM) image of the $A\beta_{42}$ peptide preparation used for ELISA plate coating, showing a heterogeneous mixture of protofibrils and fibrils.

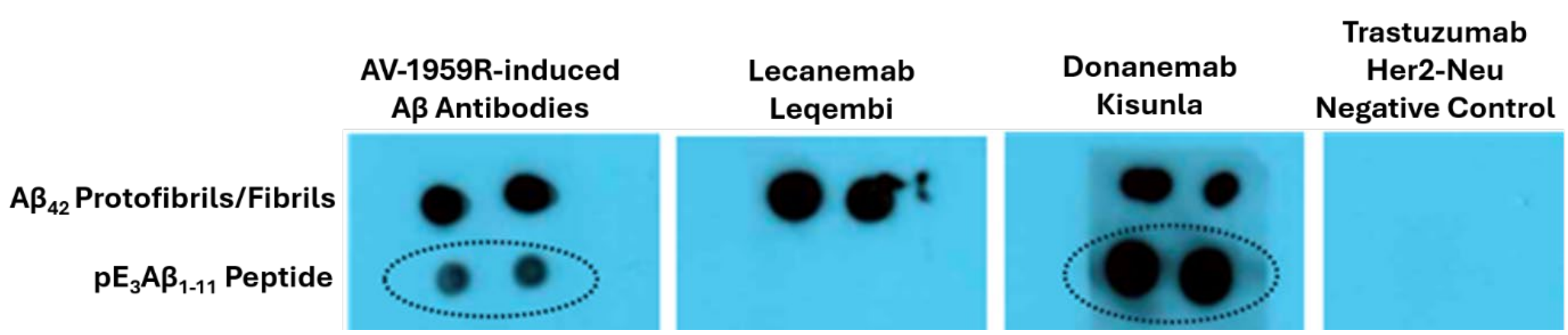

Supplementary Figure 4. Binding specificity of AV-1959R-induced antibodies compared with FDA-approved anti-amyloid monoclonal antibodies. Purified antibodies from AV-1959R-vaccinated participants were assessed by dot blot against $A\beta_{42}$ protofibrils/fibrils and pyroglutamate-modified $A\beta_{3\text{-}11}$ ($pE_3A\beta_{11}$). Vaccine-induced antibodies showed strong binding to fibrillar $A\beta_{42}$ and significant binding to $pE_3A\beta_{11}$. Lecanemab (Leqembi®) bound fibrillar $A\beta_{42}$ but not $pE_3A\beta_{11}$, whereas donanemab (Kisunla™) preferentially recognized $pE_3A\beta_{11}$. These findings demonstrate that AV-1959R elicits a polyclonal antibody repertoire that mirrors the clinically validated fibril-selective profile of lecanemab while extending coverage to pyroglutamate-modified $A\beta_{3\text{-}11}$ epitope, the N-terminal region of $pE_3A\beta_{42}$, which has been proposed to act as an early pathogenic aggregation seed.

Supplementary Table 2. Estimated values for *B1* (short-term) and *B2* (long-term) coefficients for both antibody peaks (priming and boosting).

Participant ID	B1-peak 1	B2-peak 1	B1-peak 2	B2-peak 2
**R002**	29572	6052	8003	4734
**R004**	151669	12973	28024	5088
**R005**	78964	6270	7037	6630
**R006**	30683	12044	16947	19989
**R007**	55443	8263	13229	11894
**R008**	18557	16557	2.98E-11	22518
**R025**	40138	2035	6028	2719
**R027**	49721	15202	4289	8964
**R029**	2481	298	3748	753
**R031**	79380	2467	3624	2269
**R032**	69495	8785	6107	6428
**R038**	68555	24076	10883	12945

Supplementary Table 3. Estimated time (number of days) to reach antibody titer 1:1000 after priming vaccination.

Participant ID	Days to 1:1000
**R002**	334
**R004**	458
**R005**	340
**R006**	445
**R007**	384
**R008**	497
**R025**	163
**R027**	483
**R029**	63
**R031**	191
**R032**	394
**R038**	558

P1105-00671 1 (AV-1959R)

Record Number:	4	File Name:	P1105-02-001_Release_17Oct2023_KC.dts
Temperature (°C):		Meas Date & Time:	Tuesday, October 17, 2023 11:21:00 AM
Derived Count Rate (kcps):	202.6	Solvent:	10mM phosphate pH 7.4
Intercept:	0.906	Measurement quality:	Good

Hydrodynamic Radius

Z-Average (± SD) (d.nm):	34.05 ± 25.24	Estimated MW (± SD) (KDa):	2560 ± 1.90e+03
Polydispersity Index:	0.549	%Polydispersity:	74.1
		Sample Polydispersity:	Polydisperse

Distribution Results

	Mode ± SD (nm)	%Pd	Est. MW (KDa) (Mode ± SD)*	% Intensity	% Mass	Peak Polydispersity
Peak 1:	13.54 ± 5.745	37.3	295.8 ± 192.8	34.3	98.8	Polydisperse
Peak 2:	105.7 ± 52.81	48.4	3.62e+4 ± 1.46e+4	65.7	1.2	Polydisperse
Peak 3:	0.000 ± 0.000	0	0.0 ± 0.0	0.0	0.0	

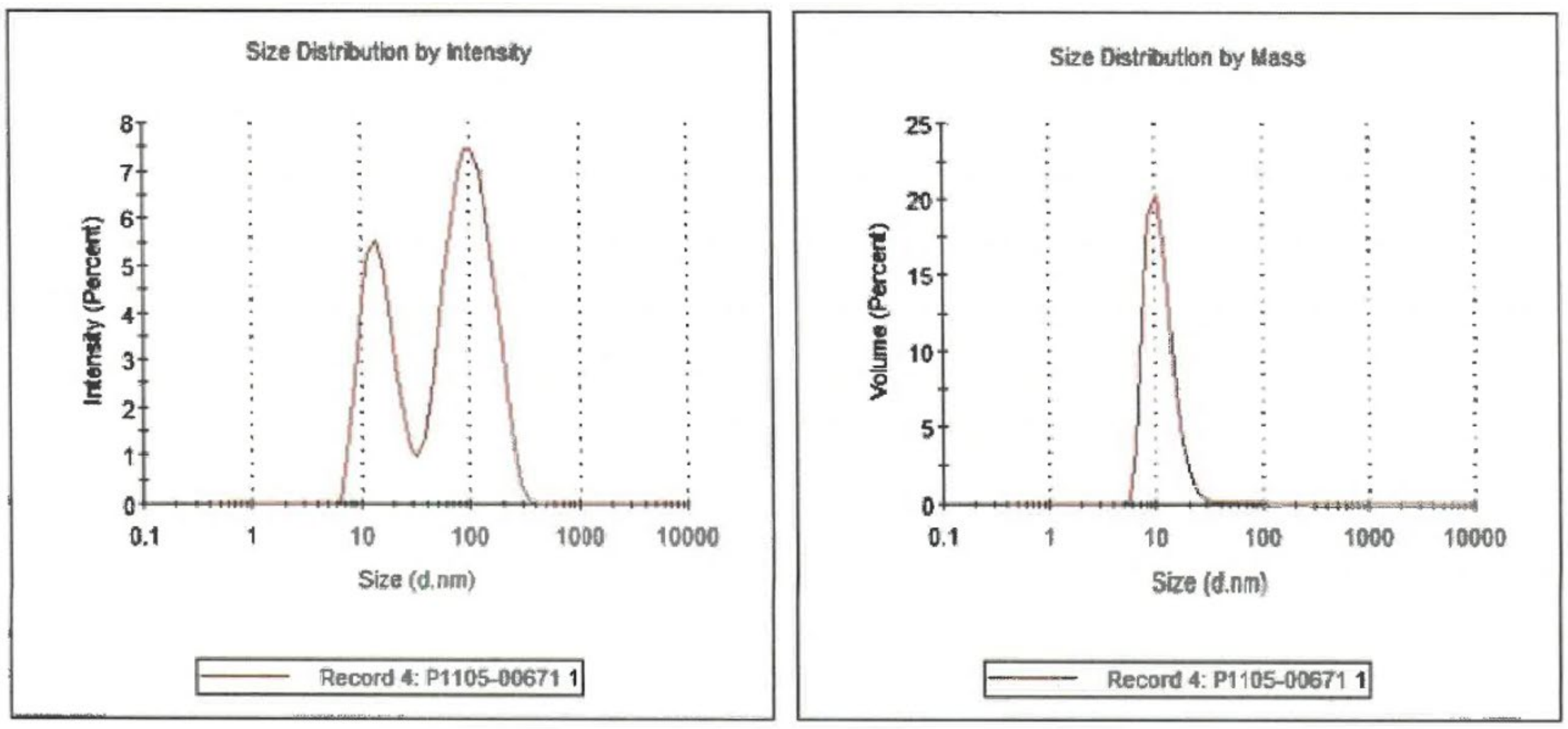

Supplementary Figure 5. DLS profile of Purified AV-1959R protein, distribution by mass and intensity. 98.8% of particles are, on average, 13.54 ± 5.745 nm in diameter with a molecular weight of 295.8 ± 192.8 kDa, suggesting a uniform size distribution. The highly aggregated population (105.7 ± 52.81 nm, MW 3.62e+4 ± 1.46e+4) accounts for only 1.2% of the protein, and the signal is not visible in the mass distribution.
